# Lys48 ubiquitination during the intraerythrocytic cycle of the rodent malaria parasite, *Plasmodium chabaudi*

**DOI:** 10.1371/journal.pone.0176533

**Published:** 2017-06-12

**Authors:** Lorena González-López, Rebeca Carballar-Lejarazú, Gerardo Arrevillaga Boni, Leticia Cortés-Martínez, Febe Elena Cázares-Raga, Abel Trujillo-Ocampo, Mario H. Rodríguez, Anthony A. James, Fidel de la Cruz Hernández-Hernández

**Affiliations:** 1 Departamento de Infectómica y Patogénesis Molecular, Centro de Investigación y de Estudios Avanzados del Instituto Politécnico Nacional (CINVESTAV-IPN), México, D.F., México; 2 Departments of Molecular Biology & Biochemistry and Microbiology & Molecular Genetics, University of California, Irvine, California, United States of America; 3 Departamento de Biomedicina Molecular, Centro de Investigación y de Estudios Avanzados del Instituto Politécnico Nacional (CINVESTAV-IPN), México, D.F., México; 4 Centro de Investigación Sobre Enfermedades Infecciosas, Instituto Nacional de Salud Pública, Cuernavaca, Morelos, México; Universidade Nova de Lisboa Instituto de Higiene e Medicina Tropical, PORTUGAL

## Abstract

Ubiquitination tags proteins for different functions within the cell. One of the most abundant and studied ubiquitin modification is the Lys48 polyubiquitin chain that modifies proteins for their destruction by proteasome. In *Plasmodium* is proposed that post-translational regulation is fundamental for parasite development during its complex life-cycle; thus, the objective of this work was to analyze the ubiquitination during *Plasmodium chabaudi* intraerythrocytic stages. Ubiquitinated proteins were detected during intraerythrocytic stages of *Plasmodium chabaudi* by immunofluorescent microscopy, bidimensional electrophoresis (2-DE) combined with immunoblotting and mass spectrometry. All the studied stages presented protein ubiquitination and Lys48 polyubiquitination with more abundance during the schizont stage. Three ubiquitinated proteins were identified for rings, five for trophozoites and twenty for schizonts. Only proteins detected with a specific anti- Lys48 polyubiquitin antibody were selected for Mass Spectrometry analysis and two of these identified proteins were selected in order to detect the specific amino acid residues where ubiquitin is placed. Ubiquitinated proteins during the ring and trophozoite stages were related with the invasion process and in schizont proteins were related with nucleic acid metabolism, glycolysis and protein biosynthesis. Most of the ubiquitin detection was during the schizont stage and the Lys48 polyubiquitination during this stage was related to proteins that are expected to be abundant during the trophozoite stage. The evidence that these Lys48 polyubiquitinated proteins are tagged for destruction by the proteasome complex suggests that this type of post-translational modification is important in the regulation of protein abundance during the life-cycle and may also contribute to the parasite cell-cycle progression.

## Introduction

Malaria is a mosquito-borne disease caused by members of the genus *Plasmodium* and it was responsible for ~300 million cases and ~580,000 deaths in 2014 [1]. The most severe forms of malaria in humans are caused by *Plasmodium falciparum*. One of the main purposes of understanding the biology of these parasites is to interrupt at least one of the replicative stages within its complicated life-cycle.

Rodent malaria species have proven useful models for investigating diverse aspects of the biology of human malaria [2]. Of these, *Plasmodium chabaudi* exhibits a synchronous intraerythrocytic cycle and other genetic and biology features that make it suitable for experimentation due to the extensive similarities between the infected mice and human infection mainly with *P*. *falciparum* [3].

The complete life-cycle of malaria parasites includes three main developmental stages, one takes place in the vector mosquito, and two in the vertebrate host in the liver and red blood cells. The latter is concluded within 48 and 24 h in *P*. *falciparum* and *P*. *chabaudi*, respectively, after a schizogonic process [3]. The parasite undergoes cell differentiation multiple times throughout its life-cycle, requiring changes in gene expression, protein synthesis and degradation [4–6]. The sequencing of *Plasmodium* genomes, together with transcriptome and proteome analyses, have shown that these processes are highly coordinated during parasite development and multiplication within erythrocytes [7, 8]. Since *Plasmodium* possesses only few DNA regulatory motifs and transcription factors, it is suggested that posttranscriptional regulation and post-translational modifications play important roles in the parasite life-cycle control [9–11]. Among these, ubiquitination, the binding of ubiquitin (Ub) units to proteins, is a major player that participates in the regulation of protein degradation, intracellular trafficking, DNA repair, cell cycle, cell differentiation, signal transduction and immune responses [5, 12–15].

Ubiquitin is a 76 amino acids (aa) protein highly conserved among all eukaryotes. Covalent attachment of Ub is carried out through a sequential enzymatic cascade catalyzed by three classes of enzymes: E1 ubiquitin-activating enzymes, E2 ubiquitin-conjugating enzymes, and E3 ubiquitin ligases, which are involved in substrate recognition [16, 17]. Ubiquitin can be attached as a single molecule (monoubiquitination), or as chains (polyubiquitination) to substrate proteins. In polyubiquitination, an isopeptide bond is formed between the Gly76 of one ubiquitin and one of seven potential lysines (Lys6, Lys11, Lys27, Lys29, Lys33, Lys48 and Lys63) present in the preceding ubiquitin [18]. Ub molecules bound through Lys48 appear to form the most abundant type and are well established as mediators of proteasomal degradation in eukaryotic cells including *Plasmodium* [12].

Three genomic loci have been identified in *P*. *falciparum* as predicted sources of ubiquitin residues. The two ubiquitin fusion proteins PfUB_S27a_ [PlasmoDB:PF3D7_1402500] and PfUB_L40_ [PlasmoDB:PF3D7_1365900] fused to ribosomal proteins S27a and L40, respectively, and the polyubiquitin gene [PlasmoDB:PF3D7_1211800] that has five direct repeats of the ubiquitin coding sequence [19]. Gene amplification and northern analyses of the polyubiquitin gene revealed that transcript levels are expressed in all the stages of the intraerythrocytic cycle with significant increases at the late trophozoite and schizont stages [19, 20]. The transcriptome profile of the intraerythrocytic cycle of *P*. *falciparum* revealed that there is a peak of expression of proteasome components during the schizont stage, indicative of a high protein turnover during this phase of the cycle [8]. In addition, proteasome inhibitors such as lactacystin, bortezomib, epoxomicin, and MLN-273 restrict parasite growth *in vitro*, supporting the hypothesis that ubiquitination could be a target to develop new drug candidates against drug-resistant strains [21–24].

*Plasmodium* uses ubiquitination to control many basic biological processes. Lysine is the second-most abundant aminoacid in *P*. *falciparum* proteins and it has been predicted that as many as 70% of the parasite proteins contain ubiquitination sites [5, 25]. Around 73 proteins are confirmed targets of ubiquitination including histone H2B [26] and actin [27]. Similar to other eukaryotic cells [25], Lys48 is the most abundantly modified, but Lys6, Lys11, Lys48 and Lys63 are also represented.

We show here, using proteomic strategies, that Lys48 ubiquitination is present in all three stages of the intraerythrocytic cycle of *P*. *chabaudi*. Schizonts exhibit high amounts of Lys48, while ring stages have the less. In addition, we identified Lys48 ubiquitinated proteins that are probably turned-off and degraded by the proteasome system. These proteins are related mainly to carbohydrate and nucleotide metabolism. The identification of these molecules provides insights on how the parasite regulates its life-cycle and the basis for the development of new anti-parasite drugs.

## Methods

### Ethical considerations

This study was approved by the CINVESTAV’s Institutional Bioethical Committee for Care and Handling of Laboratory Animals (UPEAL-Protocol 0030) following the Mexican law for humanitarian housing and management (NOM-062-ZOO-1999). Mice were anesthetized with ketamine via intraperitoneal (100 mg/kg). After this procedure, 1 ml of blood was obtained via cardiac puncture, and for death confirmation, cervical dislocation was performed (AVMA Guidelines on Euthanasia, June 2007).

### *In silico* analysis of *P*. *chabaudi* polyubiquitin protein

To identify the polyubiquitin gene and protein in different *Plasmodium* species, the gene PfUb [PlasmoDB:PF3D7_1211800; GenBank:gi|124805744] was used to blast the other *Plasmodium* species in the plasmodb database (http://plasmodb.org Version 13.0): *P*. *chabaudi* [PlasmoDB:PCHAS_061200; gi|675219656], *P*. *berghei* [PlasmoDB:PBANKA_061030; GenBank:gi|675225539], *and P*. *vivax* [PlasmoDB:PVX_084620; GenBank:gi|156101796]. The following ubiquitin genes used for sequence comparison are listed in Entrez Gene: *Homo sapiens* [GenBank:gi|601984520], *Rattus norvegicus* [GenBank:gi|8394502], *Arabidopsis thaliana* [GenBank:gi|302596003], *Drosophila melanogaster* [GenBank:gi|302595965], *Caenorhabditis elegans* [GenBank:gi|156482], *Trypanosoma brucei* [GenBank:gi|74024980], *Trichomonas vaginalis* [GenBank:gi|1101011]. ClustalOmega (www.ebi.ac.uk) was used to align sequences using the standard settings. For prediction of the ubiquitin monomer 3D structure tools at I-TASSER server (http://zhanglab.ccmb.med.umich.edu/I-TASSER/) were used and the structure with the higher confidence C-score was selected (C-score in the range of [-5, 2]) [28].

### Malaria parasites

*P*. *chabaudi* (AS) was maintained by synchronous parasitemia in 6–8 week old male mice (BALB/C strain) by weekly intraperitoneal injections with 10^6^ parasite-infected mouse red blood cells. Parasitemia was monitored by examination of Giemsa-stained blood films. Blood was collected by cardiac puncture with a heparinized syringe when mice presented a 30–40% parasitemia. Leucocytes and platelets were removed from whole blood by filtration through a cellulose powdered column (CF11, Sigma Aldrich, St. Louis, MO, USA). Infected erythrocytes were washed three times in phosphate-buffered saline (PBS), pH 7.4 and recovered by centrifugation at 1,500*g* for 5 min. Ring- trophozoite- and schizont-infected erythrocytes were separated using a Percoll (GE Healthcare, Swiss)-Sacarose (Sacarose 2.5 M 1:9 Percoll) cushion of 90%-60%. Blood was centrifuged at 18000g for 30 min. The superior phase was recovered, washed with PBS and placed over Percoll 45% and the inferior phase was recovered, washed with PBS and placed over Percoll 66%. Samples were centrifuged at 2000 rpm for 30 min and washed in PBS three times followed by centrifugation at 1,500*g* for 5 min. For protein extraction, erythrocytes were lysed with 0.15% saponin (Sigma-Aldrich, St. Louis, MO, USA), washed with PBS and stored at -70°C until use.

### RNA extraction and cDNA synthesis

RNA was extracted from *P*. *chabaudi* using TRIzol reagent (Thermo Scientific, Rockford, USA) according to the manufacturer’s instructions and cleaned using DNAse I RNase-free (Thermo Scientific). DNA absence in RNA samples was verified by PCR, using the RNA treated as template. Reverse transcription was conducted as follows: PCR mixture containing 500 μg/ml Oligo dT, RNA 10 ng/μl, dNTP Mix (10 mM each), and diethyl-pyrocarbonate-treated water was incubated for 5 min at 65°C. Then, 5X First Strand Buffer, and 0.1 M DTT were added. The PCR reaction was incubated at 42°C for 2 min, and 200 units of SuperScript II RT (Thermo Scientific) was added and incubated for 50 min at 42°C. The reaction was inactivated by heating at 70°C for 15 min.

### *P*. *chabaudi* polyubiquitin gene cloning and sequencing

The Polyubiquitin gene (PlasmoDB ID PCHAS_0612000) was amplified from cDNA from mixed stages of the intraerythrocytic cycle using the following primers (Integrated DNA Technologies): PcpUB FW (5′-ATGCAAATCTTTGTG-3′) and PcpUB RW (5′-TTAGCAACCTCCTC-3′) and the amplicon cloned in pGEM T easy (Promega, Wisconsin, USA). Plasmid DNA was purified using alkaline lysis method (Birboim, 1979) and sequenced using SP6 and T7 primers at the Langebio facilities, Cinvestav-IPN Irapuato, Mexico. After sequencing, one tandem repeat was subcloned in pGEX6P1 for recombinant protein expression by using the introduced BamHI PcUb FW (5’-GCGGATCCGCGATGCAAATCTTTGTG-3’) and EcoRI sites RV (5’-CGGAATTCCGCACCTCCTCTTAATC-3’).

### Real time quantitative RT-PCR analysis

Polyubiquitin mRNA quantification was performed on a CFX96 Real-Time PCR Detection System (Bio-Rad Laboratories, Inc., California, USA). Primers were designed using Prime3 software to amplify a 109 base pair (bp) PCR product. Primer sequences are: pCHAS-061200 FW (5’-TGATGTTGAGCCATCCGATA-3’), pCHAS-061200 RV (5’-TTCTAATTGCTTTCCAGCAAAA-3’). Polyubiquitin expression value was normalized to mRNA abundance levels and copy number of the *P*. *chabaudi* ribosomal S18 gene. The primers S18-FW (5’-ACATGGCTTTGACGGGTAAC-3’) and S18-RV (5’-GCTGCCTTCCTTAGATGTGG-3’) were used to amplify an 86 bp product. Polyubiquitin expression was quantified with SsoFast^TM^ EvaGreen SuperMix (Bio-Rad Laboratories, Inc., California, USA) using gene-specific primers in a 20 μl final reaction volume containing 10 μl of SsoFast^TM^ EvaGreen SuperMix (Bio-Rad Laboratories, Inc., California, USA), 250 nM each forward and reverse primers, and 5.0 μl cDNA sample. The amplification protocol for polyubiquitin expression consists of 30 s at 95°C, followed by 40 cycles of amplification (95°C for 5 s, 61°C for 5 s, plate read of SYBR Green I fluorescence), after which a melting-curve reaction was conducted from 65°C to 95°C with plate readings every 0.5°C. Measurements of mRNA abundance were taken in triplicate from three independent experiments and their mean used for further analyses.

### Confocal microscopy

Parasites were obtained, fixed, washed and blocked as described above. After, samples were incubated with a 1:50 dilution of rabbit anti-ubiquitin polyclonal antibody (Catalog No. ab7780, Abcam, Cambridge, UK) for 2 h at room temperature (RT). Samples were washed three times with PBS pH 7.4 by centrifugation at 1,500*g* for 5 min, and then incubated in a 1:100 dilution of Goat Anti-Rabbit IgG Alexa Fluor 488 (Catalog No. ab150077, Abcam) for 2 h RT and washed three times by centrifugation at 1,500*g* for 5 min in 1 ml of PBS. Samples were smeared on a slide and air-dried; the slide was mounted in VECTASHIELD mounting media with DAPI (Vector laboratories Inc., Burlingame, CA, USA). Confocal microscopy was performed using a Zeiss LSM 700 with a 63X/1.4 Oil DIC objective. Images were analyzed using Zen-lite Software (Zeiss, Oberkochen, Germany).

### Two-dimensional electrophoresis (2-DE)

Parasite samples were suspended in sample buffer containing 7 M urea, 2 M thiourea, 4% CHAPS, 40 mM dithiothreitol (DTT) and 2% IPG Buffer pH 3–10 (GE Healthcare), and protease- (Complete, Roche Diagnostics, Basilea, Switzerland) and phosphatase (PhosSTOP, Roche Diagnostics) inhibitor cocktails, plus 1 mM phenylmethanesulfonyl fluoride (PMSF) (Roche Diagnostics), 20 mM N-ethylmaleimide (NEM, Sigma Aldrich) to inhibit deubiquitinases (DUBs), ubiquitin-specific cysteine proteases and, 10 μM proteasome inhibitor Lactacystin (Sigma Aldrich). Lactacystin is a potent proteasome inhibitor that links covalently to the threonine hydroxyl groups on the active site of the proteasome β subunit [29]. Parasites were lysed by freeze/thaw cycles. Proteins were precipitated with 100% cold acetone and solubilized in 2-DE rehydration buffer (7 M urea, 2 M thiourea, 2% CHAPS, 65 mM DTT, and 0.5% ampholyte pH 3–10), resuspended and centrifuged at 15,000*g* for 10 min. Protein concentration was measured using 2D Quant Kit (GE Healthcare) and 150 μg of protein were applied to Immobiline^TM^ DryStrips (pH 3–10 NL, 7 cm; GE, Healthcare) and rehydrated overnight at RT. Isoelectric focusing (IEF) was performed in an Ettan IPGphor 3 System (GE Healthcare) following the manufacturer’s instructions. For the second dimension, Immobilized pH Gradient (IPG) strips were treated for 15 min in equilibration buffer (75 mM Tris-HCl, pH 8.8, 6 M urea, 29.3% glycerol, 2% SDS) with 65 mM DTT followed by 15 min in the same equilibration buffer with 67.5 mM iodoacetamide. IPG focused strips were resolved by 10% SDS-PAGE. Gels were stained with Bio-Safe colloidal Coomassie Blue (Bio-Rad Laboratories, Inc., California, USA). Images were acquired using ImageQuant LAS4000 System (GE Healthcare) [30]. Three independent biological replicates were performed to assess reproducibility of results.

### Immunoblot analyses

After the 2-DE electrophoresis, replica gels were electrotransferred onto a Hybond ECL nitrocellulose membrane (GE Healthcare, UK). For the anti-Ub immunoblot, membranes were treated in a denaturing solution (6 M guanidinium chloride, 20 mM Tris pH 7.5, 1 mM PMSF, and 5 mM 2-mercaptoethanol) for 30 min at 4°C, followed by extensive washes with Tris-buffered saline (TBS) and 0.1% Tween 20 (TBS-T) [31]. The membranes were blocked in TBS-T with 5% Bovine serum albumin (BSA) for 2 h at RT. The following antibodies were used to localize antibody-antigen complexes: mouse anti -mono and -polyubiquitin conjugates monoclonal antibody FK2 diluted 1:1000 (HRP, biotin conjugated, Cat No. BML-PW8810, Enzo, New York, USA) to reveal Streptavidin-HRP conjugated (Product Number: 21126, Thermo Scientific) diluted 1:20,000 was used. The NC membrane was stripped with Restore Western Blot Stripping Buffer (Thermo Scientific) following manufacturer's instructions and reacted with monoclonal rabbit anti-K48-linkage specific polyubiquitin antibody (Catalog No. 8081, Cell Signaling Technology) diluted 1:1000 and goat anti-rabbit IgG HRP, diluted 1:50,000 (Product No. 65–6120, Thermo Scientific) as secondary antibody.

Signal detection was performed using the Supersignal West Pico/Femto Chemiluminescent Kit (Thermo Scientific) in an ImageQuant LAS 4000 System (GE Healthcare). Assays were performed in biological triplicates. The protein spots that reacted with anti-K48-linkage polyubiquitin were excised from the replicate Coomassie stained 2-DE gels and analyzed by mass spectrometry (MS).

### Mass spectrometry for protein identification

Samples were analyzed at mass spectrometry facility of the Central Service for Experimental Research, Valencia University, Spain. Briefly, selected protein spots were in-gel digested with sequencing grade trypsin (Promega, Wisconsin, USA) as described elsewhere [32]. The digestion was stopped with 1% trifluoroacetic acid, followed by desalting and concentration using Zip Tips C-18 (Thermo Scientific) and eluted. Peptides were analyzed by nanoLC-MS/MS in a mass spectrometer nanoESI QqTOF (TripleTOF 5600 System, AB SCIEX). The triple TOF was operated in information-dependent acquisition mode, in which a 0.25^-s^ TOF MS scan from 350–1250 m/z was performed, followed by 0.05^-s^ MS/MS product ion scans from 100–1500 m/z on the 50 most intense 2+‐5+ charged ions. MS/MS spectra were analyzed using ProteinPilot Paragon algorithm or Mascot using the NCBI protein database, which compiles non-identical sequences from GenBank CDS translations, PDB, Swiss-Prot, PIR, and PRF (2014/07/02).

#### Mass spectrometry for ubiquitination residues localization

The identification of ubiquitination specific site in two antibody-identified protein spots was conducted at University of California Davis, Genome Center. Briefly, the selected protein spots were excised from a replica Coomassie stained 2-DE gels and digested as before. Peptides were analyzed by LC-MS/MS on a Q Exactive Orbitrap Mass spectrometer (Thermo Scientific) in conjunction with Proxeon Easy-nLC II HPLC (Thermo Scientific) and Proxeon nanospray source (Thermo Scientific). Tandem mass spectra were extracted and charge state deconvoluted by Proteome Discoverer software (Thermo Scientific). All MS/MS spectra were analyzed using X!Tandem (The GPM, thegpm.org; version TORNADO (2013.02.01.1)). X!Tandem was set up to search Uniprot *P*. *chabaudi* (August 2014, 15124 entries) considering the cRAP database of common laboratory contaminants (www.thegpm.org/crap; 114 entries) plus an equal number of reverse protein sequences based on predicted fragments of trypsin digestion. X!Tandem was searched with a fragment ion mass tolerance of 20 PPM and a parent ion tolerance of 20 PPM. Iodoacetamide derivative of cysteine was specified in X!Tandem as a fixed modification. Deamidation of asparagine and glutamine, oxidation of methionine and tryptophan, sulphonation of methionine, tryptophan oxidation to formylkynurenine of tryptophan, acetylation of the N-terminus, and dyglycine in lysines were specified in X!Tandem as variable modifications.

### Data processing

Since proteins were analyzed in different facilities, results were processed using different software. For protein identification, ProteinPilot default parameters were used to generate a peak list directly from TripleTOF 5600.wiff files. The ProteinPilot Paragon algorithm was used to search the NCBI protein database (2014/07/02) with the following parameters: trypsin specificity, cys‐alkylation, taxonomy restriction, and the search effort set to rapid. Proteins showing unused score >1.3 were identified with confidence ≥ 95% according to Equation 18.

For Mascot analysis, the NCBI database (2014/07/02) was used. Searches were done with tryptic specificity allowing one missed cleavage and a tolerance on the mass measurement of 100 ppm in MS mode and 0.6 Da for MS/MS ions.

Carbamidomethylation of Cys was used as a fixed modification and oxidation of Met and deamidation of Asn and Gln as variable modifications. Proteins showing scores higher than homology or significance threshold were identified with a confidence ≥ 95%.

For ubiquitinated residues identification, Scaffold (version Scaffold_4.4.1, Proteome Software Inc., Portland, OR) was used to validate MS/MS based peptide and protein identifications. Peptide identifications were accepted if they could be established at greater than 91.0% probability to achieve a False Discovery Rate (FDR) less than 1.0% by the Scaffold Local FDR algorithm [33].

## Results

### *P*. *chabaudi* polyubiquitin gene and expression

**Fig 1:**
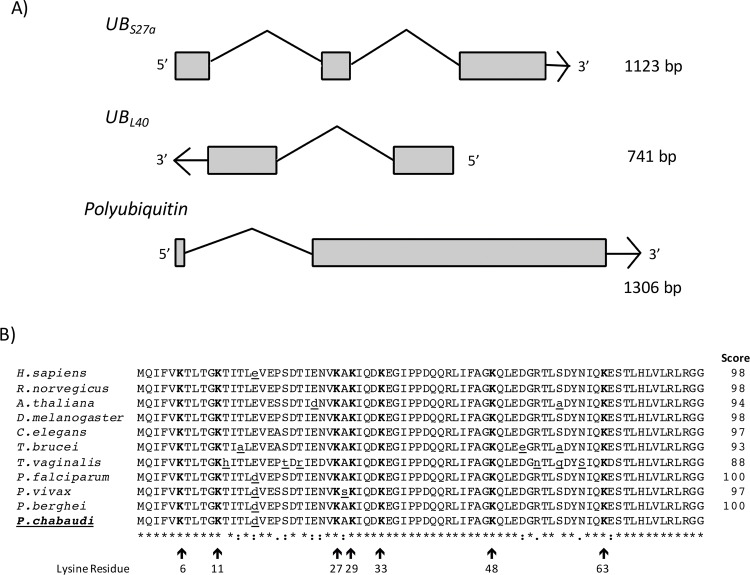
Structure of ubiquitin genes in *P*. *chabaudi* and comparison of amino acid sequences of the ubiquitin monomer among different organisms. (A) Organization of the two fusion ubiquitin genes UB_S27a_ (PCHAS_104020) and UB_L40_ (PCHAS_114120); and the polyubiquitin gene (PCHAS_061200). (B) Ubiquitin amino acids sequence alignment from divergent organisms. The identity percentage related to *P*. *chabaudi* sequence is shown at the end of each sequence. Changes in the amino acid sequence are underlined in lower case. The conserved lysine residues are shown in bold and indicated with an arrow.

*P*. *chabaudi* ubiquitin protein is predicted to be the product of two ubiquitin fusion genes, UB_S27a_ [PlasmoDB:PCHAS_104020] and UB_L40_ [PlasmoDB:PCHAS_114120], that encode a single copy of ubiquitin fused to the ribosomal protein S27a and L40, respectively, and the polyubiquitin gene [PlasmoDB:PCHAS_061200] (Fig 1A). The DNA sequence of the polyubiquitin gene is 1306 base pairs (bp) in length and contains one 5’-exon (28 bp), one intron (388 bp) and four tandemly-repeated ubiquitin moieties. Interestingly, all *Plasmodium* species have the 28 bp first exon, an intron of variable size, and 3–7 head-to-tail repeats of ubiquitin (S1A Fig).

The open reading frame (ORF) of the polyubiquitin gene encodes a pro-protein of 305 aa in length comprising four 76 aa tandem repeats with an extra amino acid residue in the last repeat probably processed by a deubiquitinating enzyme as occurs in Nedd8 where the C-terminal is processed by NEDP1 or UCHL3 [34]. The ORF, with a length of 918 bp, was cloned and sequenced (S2A Fig). The sequence obtained was compared to the one annotated in Plasmodb and was 100% identical. Each tandem repeat exhibits an identical polypeptide sequence among them; however, the nucleotide sequences of each tandem repeat presents some differences among them (S2B and S2C Fig). The ubiquitin monomer polypeptide is highly conserved through evolution, *P*. *chabaudi* has 98% identity with human sequence. Particularly, in respect to *Plasmodium* species *P*. *chabaudi* has 100% identity with *P*. *falciparum* and 97% with *P*. *vivax*. In addition, the seven lysine residues (Lys- 6, 11, 27, 29, 33, 48, and 63) subject to ubiquitination are also present in conserved positions in *P*. *chabaudi* (Fig 1B).

The I-TASSER predicted 3D structure of the ubiquitin monomer in *P*. *chabaudi* adopts the conserved ubiquitin structure (best *C*-score = 0.70) with mixed β-sheets with four of five strands and three and one half turns of α-helix. Also, a six-residue tail in the C-terminal region is present and available for activation and conjugation to other proteins (S1B Fig).

**Fig 2:**
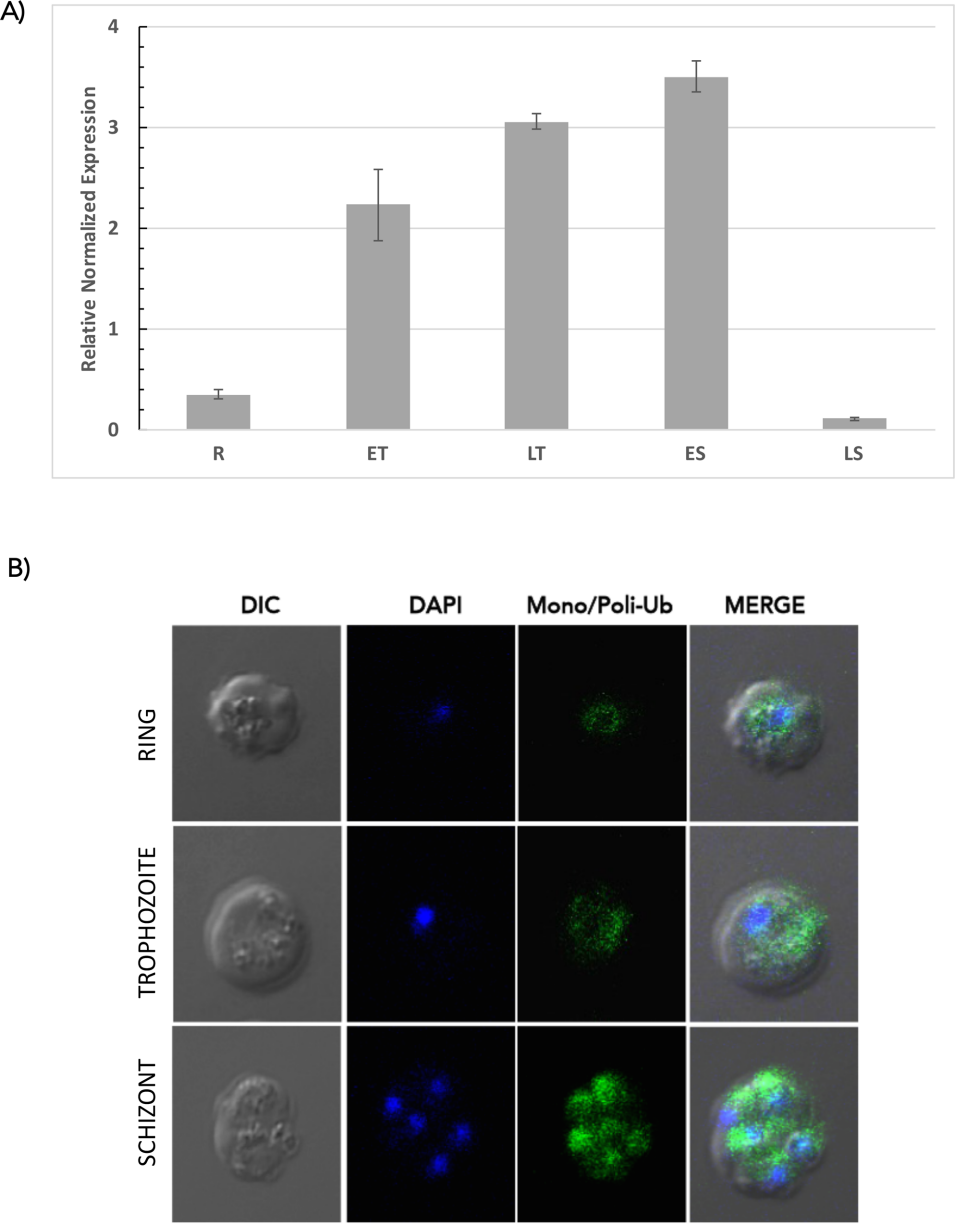
Relative expression of ubiquitin in *P*. *chabaudi*. (A)The mRNA expression of the polyubiquitin gene (PlasmoDB ID PCHAS_0612000) was determined by qRT-PCR and normalized using ribosomal gene 18S as a reference in parasites collected at different time points through the intraerythrocytic cycle (R- Ring, ET Early trophozoite, LT Late trophozoite, ES Early Schizont, and LS Late Schizont). Experiments were performed by duplicates using independent biological samples. (B) Immunofluorescence localization of total ubiquitin conjugates in parasites at their Ring, Trophozoite, or Schizont stages. The parasites were visualized under differential interference contrast (DIC) microscopy. Nuclei were stained with DAPI (*blue*), and the ubiquitin conjugates were visualized in green. Ubiquitin conjugates are present at all investigated stages.

The polyubiquitin gene expression throughout the *P*. *chabaudi* cycle was determined by qRT-PCR and ubiquitin localization (mono- and poly-ubiquitination) by florescence microscopy. Based on the results obtained from the kinetics of parasitemia in Balb/c mice, we recovered samples for RNA isolation at 6:00 am for ring stage, at 9:00 and 3:00 pm for early and late trophozoites and 6:00 pm and 9:00 pm for early and late schizonts stages (S3 Fig). We observed that the abundance of this transcript increases as the cycle progress and diminish at the end of the cycle on non-stress conditions (Fig 2A). This is similar to the results obtained for *P*. *falciparum* where the ubiquitin presents a cyclic behavior during the intraerythrocytic cycle [20]. Immunolocalization analyses showed the presence of Ub homogeneously distributed mainly through parasite cytoplasm and this signal increased as the cycle progressed (Fig 2B).

### Detection of ubiquitinated proteins by immunoblot analyses

In order to test the specific recognition of parasite Ub by the commercial monoclonal antibody, it was tested by Western-blot with the recombinant Ub protein (S4 Fig). Interestingly, the antibody only recognized Ub when it was together with Glutathione S-transferase (GST) and not when it was alone. Two-dimensional gel electrophoresis and immunoblots, using the monoclonal antibody to Mono-, Lys29, and Lys48, Lys63-linked poly-ubiquitinated proteins, were performed to identify parasites ubiquitinated proteins during the intraerythrocytic cycle. The majority of proteins were resolved between 100 kDa and 20 kDa and in a pI range between 5 and 8 (S5 Fig). However, some proteins with a higher molecular weight and with a more basic pI were also observed. The distribution of total protein among the intraerythrocytic phases remained similar among the three biological replicates.

**Fig 3:**
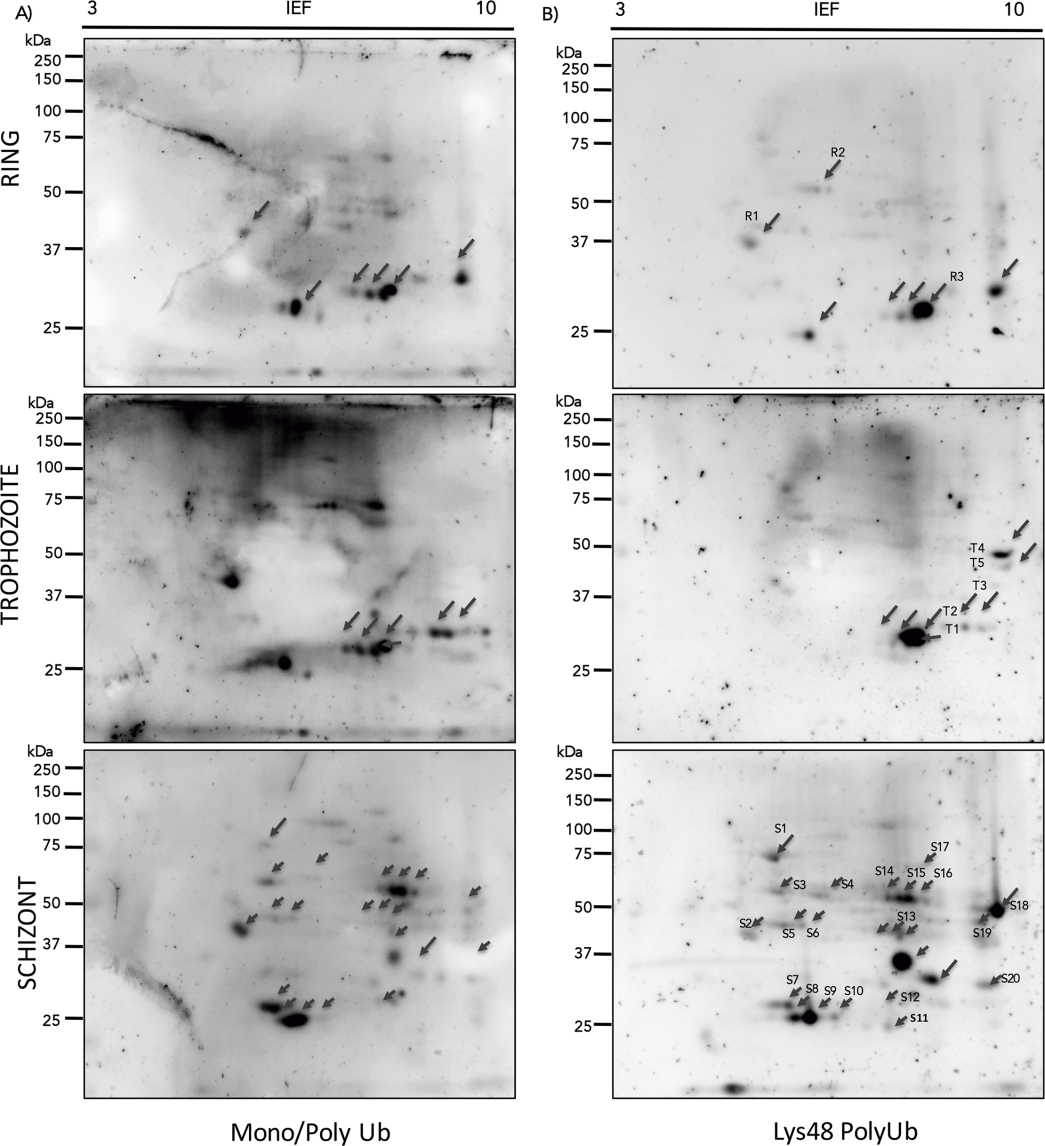
Immunoblot analyses of ubiquitinated proteins in ring-, trophozoite- and schizont-stage parasites. Replicate 2-DE gels were transferred onto nitrocellulose membranes and probed with antibodies that recognize mono- and poly-ubiquitinated proteins (panel A) and Lys48-linked polyubiquitin chains (panel B). Top panels correspond to rings, middle panels correspond to trophozoites, and bottom panels correspond to schizonts. Black arrows indicate polypeptides selected for identification by MS/MS (Table 1).

The number of ubiquitinated proteins detected by immunoblot increased as the parasite cycle progressed, consistent with our previous experiments. A total of 19, 22 and 35 protein spots were detected in the ring, trophozoite and schizont stages, respectively (Fig 3A, left side). Membranes were stripped and used to detect only polyubiquitin chains linked to Lys48 with a specific monoclonal antibody and showed the highest abundance (30 spots) of this type of polyubiquitin chain in schizont stages in comparison with seven and nine in ring and trophozoite stages, respectively (Fig 3B, right side). All of these spots were excised from replica gels (S5 Fig) and analyzed by mass spectrometry.

### Protein identification by mass spectrometry

**Fig 4:**
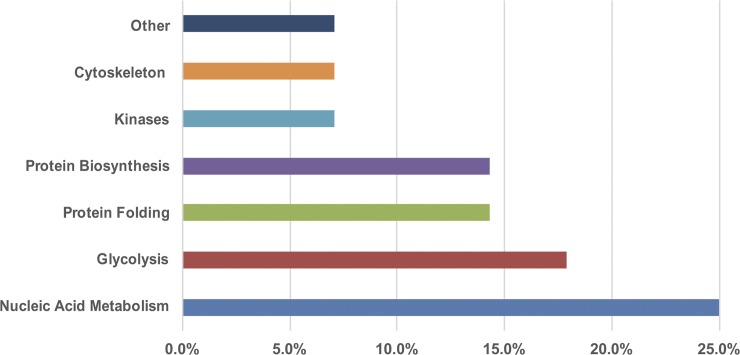
Functional annotation of the Lys48 polyubiquitinated proteins in the three intraerythrocytic stages of *P*. *chabaudi* by biological process. All proteins were detected using anti-Lys48 linked polyubiquitin chains antibodies by 2-DE/IB. Categories were obtained from the Gene Ontology/annotations of biological process at PlasmoDB.

A total of three, five, and thirty proteins bearing Lys48- linked polyubiquitin chains were identified by LC-MS/MS in ring, trophozoite, and schizont stages, respectively (Table 1 and S1 Table). The ring stage proteins (R1-R3) corresponded to actin I, elongation factor I-α (EF1α) and a hypothetical protein, respectively. The trophozoites (T1-T5) proteins corresponded to a ser/thr protein kinase (CaMK), two glyceraldehyde-3-phosphate dehydrogenase, and two EF1α) with different pI. The proteins with the highest scores in schizonts (S1-S20) were related to the metabolism of nucleic acids: purine nucleoside phosphorylase, putative (PNP); hypoxanthine-guanine-xanthine-phosphoribosyl transferase (HGPRT), and an Inosine 5’-monophosphate dehydrogenase (IMPDH). Others related to glycolysis, corresponded to phosphoglycerate kinase, hexokinase and pyruvate kinase. For protein folding, the identified proteins included the heat shock protein 70, T-complex subunits, and Hsp-organization protein (Hop). Finally, an EF1α related to protein biosynthesis was also identified. Protein functional classification was assigned using Gene Ontology resources (Fig 4).

**Table 1:** Proteins identified by MS/MS in ring-, trophozoite-, and schizont-stage parasites.

Code	Sequence Id	Accession Number (NCBI)	Protein description	Function
R1	Q4Z1L3	gi|635576196	Chain A, Crystal Structure of *P*. *berghei* Actin I with D-loop-D from muscle^1^^,^^2^	Major cytoskeleton component.
R2	K6UUT1	gi|457872484	Elongation Factor 1 alpha [*P*. *cynomolgi* strain B]^1^^,^^2^	tRNA-aminoacyl GTP-dependent binding to A site of ribosomes during protein biosynthesis.
R3	W7FNQ0	gi|579329115	Hypothetical protein PFAG_04685 [*P*. *falciparum* Santa Lucia]	Unknown.
T1	W7FQB6	gi|887563	Serine/threonine protein kinase [P. *falciparum*]	Protein kinase.
T2	Q4YRX2	gi|68067511	Glyceraldehyde-3-phosphate-dehydrogenase [*P*. *berghei* ANKA]^1^^,^^2^	Glycolysis.
T3	A0A077TNY1	gi|675222525	Glyceraldehyde-3-phosphate-dehydrogenase, hypothetical [*P*. *chabaudi chabaudi*]^1^^,^^2^	Glycolysis.
T3	Q4Z3W6	gi|68067918	Hypothetical protein [*P*. *berghei* ANKA]	Unknown.
T4	A0A077XDU3	gi|675227456	Elongation factor 1-alpha [*P*. *berghei* ANKA]^1^^,^^2^	tRNA-aminoacyl GTP-dependent binding to A site of ribosomes during protein biosynthesis.
T4	K6UX10	gi|457875124	Hypothetical protein PCYB_131280, partial [*P*. *cynomolgi* strain B]	Unknown.
T5	KUUT1	gi|457872484	Elongation Factor 1 alpha [*P*. *cynomolgi* strain B]^1^^,^^2^	tRNA-aminoacyl GTP-dependent binding to A site of ribosomes during protein biosynthesis.
S1	Q25681	gi|675219991	Heat Shock Protein, Putative [*P*. *chabaudi*] ^1^^,^^2^	Protein folding, response to stress.
S1	Q5UAH0	gi|70936729	Protein disulfide-isomerase [*P*. *chabaudi chabaudi*]	Cell redox homeostasis.
S1	Q4XNH6	gi|675220798	Heat Shock Protein 70, putative [*P*. *chabaudi*]	Protein folding, response to stress.
S1	Q4XL82	gi|70945333	Hypothetical protein, partial [*P*. *chabaudi*]	Unknown.
S2	W7FRF9	gi|124806845	Actin-1 [*P*. *falciparum*]^1^^,^^2^	Major component of the cytoskeleton.
S3	A0A077TRD4	gi|70943364	T-Complex protein 1 epsilon subunit, partial [*P*. *chabaudi chabaudi*]^1^^,^^2^	ATP binding. Protein folding. Chaperone.
S4	Q4YWP9	gi|68071207	T-Complex protein subunit beta [*P*. *berghei* ANKA]^1^^,^^2^	ATP binding. Protein folding. Chaperone.
S5	A0A024VJM9	gi|574965696	Eukaryotic initiation factor 4a, putative [*P*. *falciparum* FCH/4]	Translation initiation.
S6	A0A077TR61	gi|675222593	Eukaryotic initiation factor 4a, Putative [*P*. *chabaudi chabaudi*]	Translation initiation.
S7	A0A077TPC7	gi|675220481	fam-a protein [*P*. *chabaudi chabaudi*]	Rich tryptophan protein, immune response inductor.
S8	Q4Y6D0	gi|70951782	Purine nucleoside phosphorylase, putative (PNP) [*P*. *chabaudi*]^1^^,^^2^	Catalytic activity. Nucleoside Metabolism.
S8	W7AYM5	gi|577150680	Hypothetical protein YYG_00822 [*P*. *vinckei*]	Unknown.
S8	Q4XX86	gi|70945775	Gas41 [*P*. *chabaudi chabaudi*]	Transcription regulation.
S8	A0A077TQA0	gi|70946556	Proteasome subunit alpha type 1 [P. *chabaudi chabaudi*]	Ubiquitin-dependent protein catabolic process.
S8	A0A077TPK1	gi|70951412	Proteasome subunit beta [*P*. *chabaudi chabaudi*]	Threonine-type endopeptidase activity. Proteolysis involved in cellular protein catabolic process
S9	Q4Y6D0	gi|70951782	Purine nucleoside phosphorylase, putative (PNP) [*P*. *chabaudi*]^1^^,^^2^	Catalytic activity. Nucleoside Metabolism.
S9	Q4Y5V0	gi|70946825	Cyclin related protein [*P*. *chabaudi*]	Regulatory subunits of cyclin dependent protein kinases
S9	Q5UAH0	gi|70936729	Protein disulfide-isomerase [*P*. *chabaudi chabaudi*]	Cell redox homeostasis.
S9	Q4Y142	gi|70946556	Proteasomal subunit alpha type 1 [*P*. *chabaudi*]	Ubiquitin-dependent protein catabolic process.
S9	A0A077TPK1	gi|70951412	Proteasome beta-subunit [*P*. *chabaudi chabaudi*]	Threonine-type endopeptidase activity. Proteolysis involved in cellular protein catabolic process.
S10	Q4Y6D0	gi|70951782	Purine nucleoside phosphorylase (PNP) [*P*. *chabaudi*]^1^^,^^2^	Catabolic activity. Nucleoside metabolism.
S10	Q4Y5V0	gi|70946825	Cyclin related protein [*P*. *chabaudi*]	Regulatory subunits of cyclin dependent protein kinases.
S10	A0A077TQA0	gi|70946556	Proteasome subunidad alfa tipo 1 [*P*. *chabaudi*]	Ubiquitin-dependent protein catabolic process.
S10	A0A077TRQ3	gi|675221881	Hypoxanthine-guanine-xanthine- phosphoribosyl transferase [*P*. *chabaudi*]	Guanine phosphorybosyltransferase activity. Purine ribonucleoside salvage.
S10	W7ARB9	gi|577150919	26S proteasome non-ATPase regulatory subunit 9 [*P*. *vinckei*]	Proteasome complex.
S10	W7AXR5	gi|657009514	20S proteasome subunit alpha 4 [*P*. *vinckei vinckei*]	Threonine-type endopeptidase activity. Ubiquitin-dependent protein catabolic process.
S11	P07833	gi|123500	Hypoxanthine-guanine-xanthine- phosphoribosyl transferase [*P*. *falciparum*]^1^^,^^2^	Guanine phosphorybosyltransferase activity. Purine ribonucleoside salvage.
S12	A0A077XCD5	gi|675220823	Receptor for activated c kinase (RACK) [*P*. *chabaudi*]^1^^,^^2^	Kinase activity.
S12	A0A077YG98	gi|70938933	p1/s1 nuclease, putative [*P*. *chabaudi chabaudi*]	Endonuclease activity. DNA catabolic process.
S12	W7AR89	gi|577147954	Translation initiation factor 3 subunit I [*P*. *vinckei petteri*]	Translation initiation factor activity. Formation of translation preinitiation complex.
S12	A0A077TRQ3	gi|675221881	Hypoxanthine-guanine-xanthine- phosphoribosyl transferase [*P*. *chabaudi chabaudi*]	Guanine phosphorybosyltransferase activity. Purine ribonucleoside salvage.
S12	A0A077TKS4	gi|70944474	Pyridoxine biosynthetic enzyme pdx1 [*P*. *chabaudi chabaudi*]	Catalytic activity. Pyridoxal phosphate biosynthetic process.
S12	A0A077TTH5	gi|70951959	L-lactate dehydrogenase [*P*. *chabaudi chabaudi*]	Cellular carbohydrate metabolic process.
S13	W7FJH4	gi|124506998	Phosphoglycerate kinase OS [*P*. *falciparum*]^1^^,^^2^	Kinase. ATP binding. Glycolytic process.
S14	Q02155	gi|400025	Hexokinase OS [*P*. *falciparum*]^1^^,^^2^	Kinase. ATP binding. Glycolytic process.
S15	A0A077TN68	gi|70951516	Pyruvate kinase [*P*. *chabaudi chabaudi*]^1^^,^^2^	Carbohydrate degradation. Glycolysis.
S15	A0A077TM33	gi|70946810	Pre-mRNA-processing factor 19, putative [*P*. *chabaudi chabaudi*]	Ubiquitin-protein transferase activity. Ligase.
S15	Q4Z4S4	gi|68072367	Elongation factor 2 [*P*. *berghei* ANKA]	GTP binding. Protein biosynthesis.
S15	W7ALJ8	gi|577149624	RuvB-like protein 1 (pontin 52) [*P*. *vinckei petteri*]	DNA duplex unwinding. Nucleotide binding. DNA helicase activity.
S15	W7AHM0	gi|577149871	60S acidic ribosomal protein P0 [*P*. *vinckei petteri*]	Ribonucleoprotein. Structural constituent of ribosome.
S16	A0A077TJU2	gi|70953202	Inosine-5'-monophosphate dehydrogenase [*P*. *chabaudi chabaudi*]^1^^,^^2^	Oxidoreductase. Purine nucleotide biosynthetic process.
S16	V7PVE5	gi|83317699	Glyceraldehyde-3-phosphate-dehydrogenase [*P*. *yoelii yoelii* 17XNL]	NAD binding. Oxidoreductase activity. Glycolysis.
S16	V7PMA6	gi|82539835	Translation initiator factor E1F2 [*P*. *yoelii yoelii* 17XNL]	GTPase activity. Protein biosynthesis.
S16	A0A077TRU4	gi|70924553	Enolase [*P*. *chabaudi chabaudi*]	Lyase. Glycolytic process.
S16	A0A077TN68	gi|70951516	Pyruvate kinase [*P*. *chabaudi chabaudi*]	Carbohydrate degradation. Glycolysis.
S17	A0A077YFP9	gi|70952713	Hsp70/Hsp90 organizing protein, putative (HOP) [*P*. *chabaudi chabaudi*]^1^^,^^2^	Lyase. Glycolysis.
S17	Q4YRX2	gi|68067511	Glyceraldehyde-3-phosphate-dehydrogenase [*P*. *berghei* (Anka strain)]	NAD binding. Oxidoreductase activity. Glycolysis.
S17	A0A077THN3	gi|70947139	T-complex protein 1 [*P*. *chabaudi chabaudi*]	ATP binding. Protein folding. Chaperone.
S17	V7PNI4	gi|81177589	Elongation factor 1 alpha [*P*. *yoelii yoelii* 17XNL]	GTPase activity. Protein biosynthesis.
S18	Q00080	gi|119153	Elongation factor 1 alpha OS [*P*. *falciparum*]^1^^,^^2^	GTPase activity. Protein biosynthesis.
S19	A0A077YHS5	gi|675221037	Proliferation associated protein 2g4, putative [*P*. *chabaudi chabaudi*]^1^^,^^2^	Hydrolase.
S20	A5KC68	gi|148801687	Hypothetical protein, conserved [*P*. *vivax*]	Unknown.
S20	A0A077TLN8	gi|675221551	Acetyl-CoA synthetase, putative [*P*. *chabaudi chabaudi*]	Metabolic process, catalytic activity.

Spots were identified by MS/MS in a nano ESI qQTOF (ABSCIEX) or in a Q Exactive^TM^ Orbitrap (ThermoScientific).

^1^ Proteins identified associated with the 26 S proteasome of *P*. *falciparum* in Wang et al., 2015

^2^ Proteins identified as ubiquitinated in *P*. *falciparum* in Ponts et al., 2011

Of all the proteins identified in this study, we could find fifteen orthologues in *P*. *falciparum*, which were reported as ubiquitinated. All of them were identified in the study performed by Wang and collaborators associated with the proteasome (35) and fourteen were identified in the study performed by Ponts and collaborators [19, 25] in Ub immunoprecipitations in at least one of the 3 blood staged examined (S2 Table).

### Ubiquitin site identification

Ubiquitination sites can be identified by MS through the detection of diglycine residues (Gly-Gly) on the modified lysine. The monoisotopic mass of the diglycine adduct is 114.04 Da. These peptides are termed “ubiquitin signature peptides” [36]. We selected two spots from schizont stages to be analyzed by tandem mass to identify the specific lysine residue that was modified. From the proteins present in the spot S1 only Hsp70 (I and III) were ubiquitinated at the Lys437 and Lys222 residues, respectively. For spot S9, purine nucleoside phosphorylase was the only protein ubiquitinated at the Lys101, and Lys211 residues (Table 2 and S6 Fig).

**Table 2:** Ubiquitinated residues identified by LC/MS/MS.

Protein Number	Protein Description	Sequence^a^	Ub Residue^b^	X!Tandem^c^
S1	Heat Shock Protein, putative *(Plasmodium chabaudi)*	**(K)**SQIFTTYADNQPGVLIQVYEGER(A)	437	4.09
S1	Heat Shock Protein 70, putative *(Plasmodium chabaudi)*	(R)IINEPTAAALAFGLE**K**SDGK(V)	222	3.32
S9	Purine Nucleoside Phosphorylase, putative (*P*. *chabaudi*)	(R)AGSCGSLQPGYI**K**R(G) (K)TGGIFIVDGCPL**K**WKEGDFDEV(E)	101 211	2.35 9.32

^**a**^Peptidic sequence obtained from the mass-charge spectra. The lysine modified by ubiquitin is shown in bold and underlined.

^b^Position of the lysine within the polypeptide

^c^ Peptide Identifications were accepted if they could be established at greater than 91% probability to achieve a FDR less than 1.0% by the Scaffold Local FDR algorithm. Protein identifications were accepted if they could be established at greater than 52.0% probability to achieve an FDR less than 5.0% and contained at least 2 identified peptides.

## Discussion

This study describes protein ubiquitination during the intraerythrocytic cycle of *P*. *chabaudi*, and in particular, those that were modified at Lys48, which in other organisms triggers degradation by the 26S proteasome [12]. Ubiquitination on Lys48 is the most abundant ubiquitin linkage found in eukaryotes and we investigated which proteins might be modified by this post-translational modification in this parasite. Notably, some of the *P*. *chabaudi* experimentally detected ubiquitinated proteins were not predicted *in silico* by the available software such as *UbPred* or *UbiProber*, suggesting that the recognition elements could be species-specific.

As in other *Plasmodium* species, *P*. *chabaudi* has three genes related to ubiquitin and its polyubiquitin sequence is highly conserved. The *P*. *chabaudi* gene contains four tandem repeats of the polyubiquitin sequence in contrast to that of *P*. *falciparum* and *P*. *vivax* which have five and *P*. *berghei* which has three. A common feature in four of *Plasmodium* species analyzed is the presence of an intron, ranging from 388 to 526 bp, which is nested at the 5’ region following the first 28 bp coding exon. In contrast, in other species such as *A*. *thaliana* and *H*. *sapiens*, the intron is located in the 5’-UTR within the polyubiquitin gene. In this case, this intron mediates a high expression level by a mechanism termed intron-mediated enhancement [37]. The possible role as an expression inducer of this intron in *Plasmodium* is an interesting open question.

In higher eukaryotes as in humans, the two ubiquitin genes UbB and UbC are responsible to maintain ubiquitin homeostasis. In primates the UbB polyubiquitin genes present a constant tandem ubiquitin repeats (three) meanwhile the UbC gene is more heterogeneous with different number of tandem repeats among species [38]. In addition, UbC is thought as the most responsive gene to stress (e.g. heat shock, UV irradiation, oxidative stress) [39]. The different number of tandem repeats in *Plasmodium* among species could be related to the increase of the Ub produced, especially during stress conditions, since it would be possible to produce more ubiquitin in certain time points. In *P*. *chabaudi* the transcript level of the polyubiquitin gene exhibited a similar behavior as in *P*. *falciparum* during the intraerythrocytic cycle under no stress conditions [20], which means that the regular expression during the cycle could be a conserved mechanism.

We found by means of immunofluorescence and proteomic analysis that the ubiquitin is present during the three intraerythrocytic stages of the parasite, showing a higher abundance in schizonts. These observations are similar to those in *P*. *falciparum* [25] and in *Toxoplasma gondii*, where a significant proportion of its genome that is ubiquitinated is also transcriptionally regulated in a cell cycle-dependent manner [40]. This expression profile differs from that in other organisms where cells normally have a constitutive expression and protein conjugation increase only under stress [41, 42]. In the case of *P*. *chabaudi*, the progressive ubiquitin increase could be related to different metabolic requirements during the stages of the intraerythrocytic cycle. Regarding this, it has been proved that inhibition of proteasomes in *P*. *falciparum* during the trophozoite stage prevents DNA synthesis, and schizonts treated with proteasome inhibitors failed to egress from the erythrocyte [22]. These results support the idea that this parasite, similar to other eukaryotic cells, requires protein ubiquitination to degrade proteins that need to be recycled. This may be essential to progress during the intraerythrocytic cycle. In addition, in *P*. *falciparum* has been reported that there is a maximal induction of 29 ORFs that are predicted to encode proteasome subunits during the transition from early to mid-schizont stages. This suggest the involvement of ubiquitin-dependent protein degradation in the developmental progression of the parasite [8].

We observed in *P*. *chabaudi* that ubiquitination is present in all the asexual stages which increases from the early to mature forms and it could be related to metabolic changes throughout the intraerythrocytic cycle of the parasite, these results are comparable to those obtained in *P*. *falciparum* [25, 43].

The number of ubiquitinated proteins differed among the three parasite stages. Interestingly, the diversity of the mono- and polyubiquitin chains (Lys 29, Lys 48 and Lys 63-linked) was more evident during the ring and trophozoite stages, which could mean that protein during these stages may be ubiquitinated, not as a signal for proteolytic degradation, but to enable them to fulfill other activities, such as histone regulation, DNA repair or nuclear transport [12]. In contrast, in schizonts the Lys48 polyubiquitination increased, suggesting the recycling of proteins in preparation to egress, invasion and restart the cycle.

In *P*. *chabaudi*, we identified actin I Lys48 ubiquitinated during schizont and ring stages. Actin can be mono- or poly-ubiquitinated in muscle cells by specific ligases such as MuRF1, UbcH5 and Trim32, diminishing the levels of actin during remodeling and atrophy processes [44]. In addition, other post-translational modifications in mammals, such as arginylation, are related to actin ubiquitination for destruction through the ubiquitin proteasome system [45]. In *P*. *falciparum*, two actin genes have been so far described, *Pfactin I*, transcribed throughout the life-cycle, and *Pfactin II*, which is expressed only in the mosquito stages, including the gametocyte, gamete and sporozoite stages [46, 47].

Actin is a highly-conserved protein, although *Plasmodium* actin displays a different behavior [48]. There are contrasting reports about the presence of ubiquitinated actin during the merozoite stage, with some detecting its presence [27] while others fail to detect it during this stage [49]. Here we report that actin ubiquitination changes during the intraerythrocytic stages and it could explain this discrepancy. In addition, our results are interesting since they may indicate a role for ubiquitination regulating actin activity during the invasion and egress processes.

During the trophozoite stage, we identified as Lys48 polyubiquitinated, the ser/thr kinase related to the calcium/calmodulin dependent protein kinase (CaMK). The intraerythrocytic expression profiles in *P*. *falciparum* show that the mRNA of this kinase peaks during merozoite and ring stages and the expression decays during the trophozoite stage [50].

Several proteins that we identified to be modified by Lys48 linked polyubiquitin at the schizont stage were those that are highly abundant during the trophozoite stage such as glycolytic enzymes. Glucose is the main energy source of *Plasmodium* during the asexual blood stage, and the enzymes related to its metabolism are increased around 70-fold during the trophozoite stage [51]. After this point, parasite metabolism decreases during maturation.

We identified by 2-DE followed by immunoblot several ubiquitinated species corresponding to EF-1 α. The two genes encoding EF-1 α in *P*. *knowlesi* and *P*. *berghei* are expressed in all asexual stages except the mature schizont-stage [52]. In mammalian cells, eEF1A1 transcription is up-regulated in growing cells and down-regulated in resting cells to reduce the level of translation [53]. EF-1 α also can promote the degradation of N α-acetylated proteins and can interact with the proteasome for the degradation of damaged nascent proteins. eEF1A can be ubiquitinated and degraded by the 26S proteasome [54–56]. The abundance of these spots observed in Coomassie blue stained gels changed suggesting that the molecule may have different post-translational modifications and the dynamics of the molecule should be analyzed particularly, because it participates in many cellular functions, including protein quality control, co-translational degradation, actin bundling, nuclear export among others [57].

Parasite DNA replication takes place during the trophozoite stage. *Plasmodium* requires purines and pyrimidines for DNA/RNA synthesis and other metabolic pathways for this purpose during its exponential multiplication in the mammalian hosts. Since *Plasmodium* is a purine auxotroph, large quantities of RNA and DNA precursors must be salvaged from the mammalian host [58]. We identified at least three Lys48 ubiquitinated proteins of the purine salvage pathway, HGPRT, IMPDH and a protein annotated as PNP during the schizont stage. These enzymes are involved in the nucleotide production used in nucleic acid synthesis [58]. The first one is a key enzyme for the production of monophosphorylated nucleotide and inorganic pyrophosphate. Another enzyme found downstream in the same purine salvage pathway, inosine 5’-monophosphate dehydrogenase, is the enzyme that converts inosine monophosphate (IMP) into xanthine monophosphate (XMP) in order to obtain guanylate nucleotides [58, 59]. We identified PNP, which is responsible for the phosphorolysis of nucleosides into the corresponding nucleobases and ribose 1-phosphate [60]. In *Plasmodium*, it has been stated that the PNP is a key enzyme that catalyzes the phosphorolysis, mainly of inosine, to produce hypoxanthine and ribose 1-phosphate [61]. At least three proteins of this pathway were identified as Lys48 ubiquitinated during the schizont stage. Since these proteins are related to the DNA and RNA synthesis that takes place mainly during the trophozoite stage, it is reasonable to propose that after this highly metabolic stage, these proteins may be downregulated or degraded by a proteasome-dependent mechanism. These results are relevant because the purine nucleoside monophosphates salvage pathway has been studied as a therapeutic target because of its differences with the mammalian pathway [62, 63]. Interestingly, subunits of the proteasome were also identified in the same spots where the PNP was identified.

Hsp70 and Hsp70/Hsp90 organizing protein (Hop) were identified Lys48-polyubiquitinated in this work. Many roles have been allocated to heat-shock proteins, including the canonical heat-shock response, assembly and disassembly of multiprotein complexes, signal transduction and they also work in ubiquitination systems to degrade proteins that cannot be refolded [64]. When the chaperons carry no client proteins, the E3 ubiquitin-ligase CHIP catalyses their polyubiquitination and subsequent proteasomal degradation [65]. Chaperones in *Plasmodium* are central for growth and development, and at least six Hsp70 homologs have been identified, all of them exhibiting different roles within the cell [66]. PfHsp70-I is the major cytosolic chaperone in intraerythrocytic stages and is the component of the PfHsp90-PfHsp70 multi-chaperone machinery. The activity and co-interaction of this complex is modulated by the Hsp70-Hsp90 organizing protein (Hop) and although there is scarce information about the role of this protein in *Plasmodium*, this co-chaperone was found overexpressed at the trophozoite stage and in association with both chaperones [67]. Interestingly, *P*. *chabaudi* Hsp70 possesses the C-terminal EEVD motif present in eukaryotic cytosolic Hsp70s, and thus this protein potentially may interact with Hop, an essential co-chaperone for protein folding in other models. It has been demonstrated that mRNA and this co-chaperone are highly abundant in primary human cancers, supporting its role in a pro-folding environment [68].

Since Lys48 linked-polyubiquitin tag proteins for destruction by the proteasome, we selected to Hsp70 and PNP proteins in order to identify the specific site of ubiquitination. For Hsp70, we could identify two different Hsp70 (I and III) with only one lysine ubiquitinated for each one and for PNP we identified two different lysine residues within the protein (Table 2 and S1 Fig). It was previously documented in *P*. *falciparum* that Hsp70-I, and PNP proteins and transcripts decay at the end of the parasite asexual cycle, during the schizont stage [69]. Based on the transcriptome of the intraerythrocytic cycle of *P*. *falciparum* it was found that the mRNA levels of 16 enzymes of the nucleotide synthesis, including PNP, peaked at approximately 18–22 hpi (at the trophozoite stage) and then rapidly decline [8]. Based on the information about both proteins Hsp70 and PNP, we propose that these are post-translationally regulated by Lys48 linked ubiquitination. Interestingly, most of the proteins found in this work were associated with the proteasome or even ubiquitinated in *P*. *falciparum* [25, 35]. This suggest that the Lys48 linked-polyubiquitination may be a conserved process within *Plasmodium* species.

## Conclusions

Ubiquitination is an important biological process for intracellular trafficking, DNA repair, cell cycle, differentiation, signal transduction and immune response. In this work we show that the ubiquitination is carried out during the entire *P*. *chabaudi* intraerythrocytic cycle, mainly during the schizont stage. In particular, the Lys48 polyubiquitin chain, which marks proteins for degradation, was observed mainly during the schizont stage, which indicate that this process is implicated in protein degradation before parasite release.

The proteins identified in this study are involved in different processes such as DNA/RNA synthesis, protein folding, and metabolism, and their expression change according to life-cycle progress. The evidence that the proteins identified are ubiquitinated for destruction in the proteasome complex suggests that this type of post-translational modification is important in the regulation of protein abundance during the cycle and also may contribute to the parasite cycle progression.

Since proteasome inhibitors have profound effects on *Plasmodium* development, obtaining new information on the parasite ubiquitination process could be used for new drug design.

## Supporting information

S1 Table Proteins identified by MS/MS in ring-, trophozoite-, and schizont-stage parasites. (DOCX)

S2 Table Ubiquitinated proteins found in other studies performed in *P*. *falciparum*. (DOCX)

S1 Fig Comparison of polyubiquitin genes of different *Plasmodium* species and ubiquitin secondary structure. (A) Schematic representation of the polyubiquitin gene in *P*. *falciparum* (PF3D7_1211800), *P*. *vivax* (PVX_084620), *P*. *chabaudi* (PCHAS_061200) and *P*. *berghei* (PBANKA_061030). The first exon of each species has a length of 28 bp. The intron has a length of 526 bp, 492 bp, 388 bp, and 436 bp for *P*. *falciparum*, *P*. *vivax*, *P*, *chabaudi*, and *P*. *berghei*, respectively. (B) Comparison of the secondary structure of the ubiquitin molecule in *H*. *sapiens* (blue, PDB code 1UBQ) and the *P chabaudi* structure (green) modelled by I-Tasser (C-score = 0.70). Both structures present a globular conformation with five beta-sheets and one alpha helix. Both sequences are structurally similar and can be overlapped.
(TIF)

S2 Fig *Plasmodium chabaudi* polyubiquitin sequence. (A) Sequence of the ORF of the polyubiquitin gene (B) Alignment of the DNA sequence of the four tandem repeats of the polyubiquitin gene using Clustal Omega (C) Alignment of the aminoacids sequence of the four tandem repeats of the Ubiquitin polypeptides using Clustal Omega.
(DOCX)

S3 Fig Parasitemia of *P*. *chabaudi* in male BALB/c mice. **A)** Male BALB/c mice of 6–8 weeks were infected with 1 x 10^6^ parasites. Every 3 hours the parasite stages were counted. Counts were performed by triplicates, and are expressed as percentage of infected erythrocytes. **B) Different intraerythrocytic stages of *P*. *chabaudi* obtained after Percoll purification**. Smears of the parasites purified with Percoll-Sucrose gradients were stained with 20% Giemsa. a) Ring stage (3 am); b-d Trophozoite stage (obtained at 9 am, 3 pm, 7 pm, respectively); e-f) Schizont stage (obtained at 6 pm and 9 pm, respectively).
(TIF)

S4 Fig Immunoblot analysis of PcUb recombinant protein against anti-Ubiquitin monoclonal antibody. Specificity of the anti-Ubiquitin monoclonal antibody was tested with the Ubiquitin monomer recombinant protein of *P*. *chabaudi*. Lane 1–5. Successive fractions after chromatographic elution of GST-PcUb.
(TIF)

S5 Fig 2-DE gel electrophoretic analysis of *P*. *chabaudi* rings, trophozoites, and schizont proteins. Proteins of rings (A), trophozoites (B), and schizonts (C) were resolved on Immobiline DryStrips (pH 3–10, NL 7 cm) and second dimension was performed using 10% SDS-PAGE. Gels were stained with Coomassie Blue G250. Molecular mass markers (kDa) are indicated to the left. Isoelectric points (pI) are indicated at the top. White arrows indicate the polypeptides selected for MS identification.
(TIF)

S6 Fig Identification of the ubiquitin modified residue by MS/MS. After trypsin digestion, the peptide that contains the post-translational modification has a diglycline remnant covalently attached to a lysine residue that is resistant to the trypsin proteolysis (36). On each figure (A-D) is shown the peptide sequence produced by the trypsin proteolysis with the ion mass (type b and y ions) and with the diglycine modification (top figure) and the fragmentation pattern (MS/MS spectra) acquired for the peptide sequence construction. (A) Identification of the lysine modification for a heat-shock protein, putative; (B) Identification of the lysine modification for a heat-shock protein 70; (C-D) Identification of the lysines modifications for the uridine phosphorylase/purine nucleoside phosphorylase.
(TIF)
