# Supplementary material for: Lys48 ubiquitination during the intraerythrocytic cycle of the rodent malaria parasite, Plasmodium chabaudi
Source: PLoS One. 2017 Jun 12;12(6):e0176533. doi: 10.1371/journal.pone.0176533 (PMC5467854; doi:10.1371/journal.pone.0176533)
Supplement: S1 Table — (DOCX) [file pone.0176533.s001.docx]

**S6. Proteins identified by MS/MS in ring-, trophozoite-, and schizont-stage parasites**

| Side code | Sequence Id | Accession Number (NCBI) | Protein description | Function | Theor./Exp. Mr^a^ | Theor./ exp pI^b^ | Unused^c^/  Score^d^/  Total Spectrum Count^e^ | % Cov.^f^ |
| --- | --- | --- | --- | --- | --- | --- | --- | --- |
| R1 | Q4Z1L3 | gi\|635576196 | Chain A, Crystal Structure of *P. berghei* Actin I with D-loop-D from muscle | Major cytoskeleton component. | 41.9/40 | 5.0/5.0 | 3 ^c^ | 51 |
| R2 | K6UUT1 | gi\|457872484 | Elongation Factor 1 alpha [*P. cynomolgi* strain B] | tRNA-aminoacyl GTP-dependent binding to A site of ribosomes during protein biosynthesis. | 48.9/60 | 9.6/6.0 | 2^c^ | 10 |
| R3 | W7FNQ0 | gi\|579329115 | Hypothetical protein PFAG_04685 [*P. falciparum* Santa Lucia] | Unknown. | 168.8/28 | 6.6/7.8 | 1.7 ^c^ | 9 |
| T1 | W7FQB6 | gi\|887563 | Serine/threonine protein kinase [P. *falciparum*] | Protein kinase. | 90.8/28 | 10.2/7.8 | 2 ^c^ | 9 |
| T2 | Q4YRX2 | gi\|68067511 | Glyceraldehyde-3-phosphate-dehydrogenase [*P. berghei* ANKA] | Glycolysis. | 36.6/29 | 7.9/8.0 | 4 ^c^ | 7 |
| T3 | A0A077TNY1 | gi\|675222525 | Glyceraldehyde-3-phosphate-dehydrogenase, hypothetical [*P. chabaudi chabaudi*] | Glycolysis. | 36.6/33 | 7.7/8.5 | 6.9 ^c^ | 22 |
| T3 | Q4Z3W6 | gi\|68067918 | Hypothetical protein [*P. berghei* ANKA] | Unknown. | 79.7/33 | 8.5/8.5 | 1.95 ^c^ | 3 |
| T4 | A0A077XDU3 | gi\|675227456 | Elongation factor 1-alpha [*P. berghei* ANKA] | tRNA-aminoacyl GTP-dependent binding to A site of ribosomes during protein biosynthesis. | 48.9/45 | 9.6/9.6 | 12.8 ^c^ | 25 |
| T4 | K6UX10 | gi\|457875124 | Hypothetical protein PCYB_131280, partial [*P. cynomolgi* strain B] | Unknown. | 80.3/45 | 9.8/9.6 | 2 ^c^ | 2 |
| T5 | KUUT1 | gi\|457872484 | Elongation Factor 1 alpha [*P. cynomolgi* strain B] | tRNA-aminoacyl GTP-dependent binding to A site of ribosomes during protein biosynthesis. | 48.0/49 | 9.6/9.6 | 35.7 ^c^ | 61 |
| S1 | Q25681 | gi\|675219991 | Heat Shock Protein, Putative [*P. chabaudi*] | Protein folding, response to stress. | 74/ 75 | 5.3/5.3 | 538^e^ | 54 |
| S1 | Q5UAH0 | gi\|70936729 | Protein disulfide-isomerase [*P. chabaudi chabaudi*] | Cell redox homeostasis. | 55/75 | 5.5/5.3 | 93^e^ | 58 |
| S1 | Q4XNH6 | gi\|675220798 | Heat Shock Protein 70, putative [*P. chabaudi*] | Protein folding, response to stress. | 73.1/75 | 6.6/5.3 | 41^e^ | 49 |
| S1 | Q4XL82 | gi\|70945333 | Hypothetical protein, partial [*P. chabaudi*] | Unknown. | 14.1/75 | 10.5/5.3 | 6 ^e^ | 2 |
| S2 | W7FRF9 | gi\|124806845 | Actin-1 [*P. falciparum*] | Major component of the cytoskeleton. | 42/45 | 5.2/5.0 | 547^d^ | 58 |
| S3 | A0A077TRD4 | gi\|70943364 | T-Complex protein 1 epsilon subunit, partial [*P. chabaudi chabaudi*] | ATP binding. Protein folding. Chaperone. | 59/56 | 5.5/5.5 | 10^c^ | 19 |
| S4 | Q4YWP9 | gi\|68071207 | T-Complex protein subunit beta [*P. berghei* ANKA] | ATP binding. Protein folding. Chaperone. | 59.1/53 | 6.4/6.0 | 5.7 ^c^ | 8 |
| S5 | A0A024VJM9 | gi\|574965696 | Eukaryotic initiation factor 4a, putative [*P. falciparum* FCH/4] | Translation initiation. | 45.3/43 | 5.4/5.5 | 23.23 ^c^ | 42 |
| S6 | A0A077TR61 | gi\|675222593 | Eukaryotic initiation factor 4a, Putative [*P. chabaudi chabaudi*] | Translation initiation. | 45.3/43 | 5.8/5.8 | 23.63 ^c^ | 45 |
| S7 | A0A077TPC7 | gi\|675220481 | fam-a protein [*P. chabaudi chabaudi*] | Rich tryptophan protein, immune response inductor. | 32.3/30 | 6.2/5.6 | 28.15 ^c^ | 74 |
| S8 | Q4Y6D0 | gi\|70951782 | Purine nucleoside phosphorylase, putative (PNP) [*P. chabaudi*] | Catalytic activity. Nucleoside  Metabolism. | 27/26 | 5.8/5.7 | 70.8^c^ | 89 |
| S8 | W7AYM5 | gi\|577150680 | Hypothetical protein YYG_00822 [*P. vinckei*] | Unknown. | 29/26 | 5.6/5.7 | 15.5^c^ | 29 |
| S8 | Q4XX86 | gi\|70945775 | Gas41 [*P. chabaudi chabaudi*] | Transcription regulation. | 26/26 | 8.1/5.7 | 8.9^c^ | 39 |
| S8 | A0A077TQA0 | gi\|70946556 | Proteasome subunit alpha type 1 [P*. chabaudi chabaudi*] | Ubiquitin-dependent protein catabolic process. | 28/26 | 5.9/5.7 | 6.86^c^ | 26 |
| S8 | A0A077TPK1 | gi\|70951412 | Proteasome subunit beta [*P. chabaudi chabaudi*] | Threonine-type endopeptidase  activity. Proteolysis involved in  cellular protein catabolic process | 30/26 | 6.2/5.7 | 6.7^c^ | 26 |
| S9 | Q4Y6D0 | gi\|70951782 | Purine nucleoside phosphorylase, putative (PNP) [*P. chabaudi*] | Catalytic activity. Nucleoside  Metabolism. | 27/26 | 5.8/5.8 | 534^e^ | 52 |
| S9 | Q4Y5V0 | gi\|70946825 | Cyclin related protein [*P. chabaudi*] | Regulatory subunits of cyclin  dependent protein kinases | 25/26 | 4.8/5.8 | 110 ^e^ | 56 |
| S9 | Q5UAH0 | gi\|70936729 | Protein disulfide-isomerase [*P. chabaudi chabaudi*] | Cell redox homeostasis. | 55/26 | 5.5/5.8 | 28 ^e^ | 32 |
| S9 | Q4Y142 | gi\|70946556 | Proteasomal subunit alpha type 1 [*P. chabaudi*] | Ubiquitin-dependent protein catabolic process. | 29/26 | 5.9/5.8 | 22 ^e^ | 35 |
| S9 | A0A077TPK1 | gi\|70951412 | Proteasome beta-subunit [*P. chabaudi chabaudi*] | Threonine-type endopeptidase activity. Proteolysis involved in cellular protein catabolic process. | 30/26 | 6.2/5.8 | 11 ^e^ | 21 |
| S10 | Q4Y6D0 | gi\|70951782 | Purine nucleoside phosphorylase (PNP) [*P. chabaudi*] | Catabolic activity.  Nucleoside metabolism. | 27/26 | 5.8/6.3 | 76.01^c^ | 95 |
| S10 | Q4Y5V0 | gi\|70946825 | Cyclin related protein [*P. chabaudi*] | Regulatory subunits of cyclin  dependent protein kinases. | 25/26 | 4.8/6.3 | 50.9^c^ | 72 |
| S10 | A0A077TQA0 | gi\|70946556 | Proteasome subunidad alfa tipo 1 [*P. chabaudi*] | Ubiquitin-dependent protein catabolic process. | 29/26 | 5.9/6.3 | 29.2^c^ | 70 |
| S10 | A0A077TRQ3 | gi\|675221881 | Hypoxanthine-guanine-xanthine- phosphoribosyl transferase [*P. chabaudi*] | Guanine phosphorybosyltransferase activity. Purine ribonucleoside salvage. | 27/26 | 6.2/6.3 | 24.9^c^ | 63 |
| S10 | W7ARB9 | gi\|577150919 | 26S proteasome non-ATPase regulatory subunit 9 [*P. vinckei*] | Proteasome complex. | 26/26 | 5.6/6.3 | 9.5^c^ | 48 |
| S10 | W7AXR5 | gi\|657009514 | 20S proteasome subunit alpha 4 [*P. vinckei vinckei*] | Threonine-type endopeptidase activity. Ubiquitin-dependent protein catabolic process. | 27/26 | 6.4/6.3 | 8.8^c^ | 48 |
| S11 | P07833 | gi\|123500 | Hypoxanthine-guanine-xanthine- phosphoribosyl transferase  [*P. falciparum*] | Guanine phosphorybosyltransferase activity. Purine ribonucleoside salvage. | 27/25 | 7.6/7.3 | 86^d^ | 18 |
| S12 | [A0A077XCD5](http://www.uniprot.org/uniprot/A0A077XCD5) | gi\|675220823 | Receptor for activated c kinase (RACK) [*P. chabaudi*] | Kinase activity. | 36/30 | 6.3/7.2 | 54.2^c^ | 75 |
| S12 | A0A077YG98 | gi\|70938933 | p1/s1 nuclease, putative [*P. chabaudi chabaudi*] | Endonuclease activity. DNA catabolic process. | 38/30 | 6.2/7.2 | 40.5^c^ | 72 |
| S12 | W7AR89 | gi\|577147954 | Translation initiation factor 3 subunit I [*P. vinckei petteri*] | Translation initiation factor activity. Formation of translation preinitiation complex. | 37/30 | 6.3/7.2 | 24.3^c^ | 49 |
| S12 | A0A077TRQ3 | gi\|675221881 | Hypoxanthine-guanine-xanthine- phosphoribosyl transferase  [*P. chabaudi chabaudi*] | Guanine phosphorybosyltransferase activity. Purine ribonucleoside salvage. | 27/30 | 6.2/7.2 | 16.7^c^ | 54 |
| S12 | A0A077TKS4 | gi\|70944474 | Pyridoxine biosynthetic enzyme pdx1 [*P. chabaudi chabaudi*] | Catalytic activity. Pyridoxal phosphate biosynthetic process. | 33/30 | 7.0/7.2 | 8.5^c^ | 27 |
| S12 | A0A077TTH5 | gi\|70951959 | L-lactate dehydrogenase [*P. chabaudi chabaudi*] | Cellular carbohydrate metabolic process. | 34/30 | 7.7/7.2 | 6.7^c^ | 29 |
| S13 | W7FJH4 | gi\|124506998 | Phosphoglycerate kinase OS [*P. falciparum*] | Kinase. ATP binding. Glycolytic process. | 46/43 | 7.6/7.4 | 170^d^ | 18 |
| S14 | Q02155 | gi\|400025 | Hexokinase OS [*P. falciparum*] | Kinase. ATP binding. Glycolytic process. | 56/50 | 7.0/7.3 | 127^d^ | 31 |
| S15 | A0A077TN68 | gi\|70951516 | Pyruvate kinase [*P. chabaudi chabaudi*] | Carbohydrate degradation. Glycolysis. | 56/50 | 6.8/7.6 | 61.9^c^ | 73 |
| S15 | A0A077TM33 | gi\|70946810 | Pre-mRNA-processing factor 19, putative [*P. chabaudi chabaudi*] | Ubiquitin-protein transferase activity. Ligase. | 57/50 | 6.8/7.6 | 23^c^ | 41 |
| S15 | Q4Z4S4 | gi\|68072367 | Elongation factor 2 [*P. berghei* ANKA] | GTP binding. Protein biosynthesis. | 93/50 | 6.7/7.6 | 9.9^c^ | 11 |
| S15 | W7ALJ8 | gi\|577149624 | RuvB-like protein 1 (pontin 52) [*P. vinckei petteri*] | DNA duplex unwinding. Nucleotide binding. DNA helicase activity. | 53/50 | 6.7/7.6 | 7.4^c^ | 18 |
| S15 | W7AHM0 | gi\|577149871 | 60S acidic ribosomal protein P0 [*P. vinckei petteri*] | Ribonucleoprotein. Structural constituent of ribosome. | 35/50 | 7.7/7.6 | 4.9^c^ | 22 |
| S16 | A0A077TJU2 | gi\|70953202 | Inosine-5'-monophosphate dehydrogenase [*P. chabaudi chabaudi*] | Oxidoreductase. Purine nucleotide biosynthetic process. | 56/50 | 7.5/7.9 | 48.4^c^ | 67 |
| S16 | V7PVE5 | gi\|83317699 | Glyceraldehyde-3-phosphate-dehydrogenase [*P. yoelii yoelii* 17XNL] | NAD binding. Oxidoreductase activity. Glycolysis. | 37/50 | 7.7/7.9 | 8.1^c^ | 30 |
| S16 | V7PMA6 | gi\|82539835 | Translation initiator factor E1F2 [*P. yoelii yoelii* 17XNL] | GTPase activity. Protein biosynthesis. | 51/50 | 8.0/7.9 | 5.4^c^ | 15 |
| S16 | A0A077TRU4 | gi\|70924553 | Enolase [*P. chabaudi chabaudi*] | Lyase. Glycolytic process. | 49/50 | 6.5/7.9 | 4^c^ | 21 |
| S16 | A0A077TN68 | gi\|70951516 | Pyruvate kinase [*P. chabaudi chabaudi*] | Carbohydrate degradation. Glycolysis. | 56/50 | 6.8/7.9 | 4.5^c^ | 16 |
| S17 | A0A077YFP9 | gi\|70952713 | Hsp70/Hsp90 organizing protein, putative (HOP) [*P. chabaudi chabaudi*] | Lyase. Glycolysis. | 66/70 | 7.5/8.5 | 93^c^ | 79 |
| S17 | Q4YRX2 | gi\|68067511 | Glyceraldehyde-3-phosphate-dehydrogenase [*P. berghei* (Anka strain)] | NAD binding. Oxidoreductase activity. Glycolysis. | 37/50 | 7.9/8.5 | 14.3^c^ | 52 |
| S17 | A0A077THN3 | gi\|70947139 | T-complex protein 1 [*P. chabaudi chabaudi*] | ATP binding. Protein folding. Chaperone. | 60/50 | 7.4/8.5 | 11.3^c^ | 15 |
| S17 | V7PNI4 | gi\|81177589 | Elongation factor 1 alpha [*P. yoelii yoelii* 17XNL] | GTPase activity. Protein biosynthesis. | 49/50 | 9.6/8.5 | 3.2^c^ | 5 |
| S18 | Q00080 | gi\|119153 | Elongation factor 1 alpha OS [*P. falciparum*] | GTPase activity. Protein biosynthesis. | 49/49 | 9.3/9.6 | 287^d^ | 47 |
| S19 | A0A077YHS5 | gi\|675221037 | Proliferation associated protein 2g4, putative [*P. chabaudi chabaudi*] | Hydrolase. | 42.5/43 | 8.2/9.0 | 5.73^c^ | 19 |
| S20 | A5KC68 | gi\|148801687 | Hypothetical protein, conserved [*P. vivax*] | Unknown. | 233.7/32 | 7.6/9.1 | 1.7 ^c^ | 8 |
| S20 | A0A077TLN8 | gi\|675221551 | Acetyl-CoA synthetase, putative [*P. chabaudi chabaudi*] | Metabolic process, catalytic activity. | 102.3/32 | 8.1/9.1 | 1.7 ^c^ | 7 |

Spots were identified by MS/MS in a nano ESI qQTOF (ABSCIEX) or in a Q Exactive^TM^ Orbitrap (ThermoScientific).

^a^Theoretical/Experimental mass (Mr).

^b^Theoretical/Experimental pI.

^c^ MS/MS score of proteins identified by Protein Pilot. Proteins showing unused score >1.3 were identified with confidence ≥ 95%.

^d^ MS/MS score of proteins identified by Mascot program. Scores greater than 70 are significant (p<0.05).

^e^ MS/MS score of proteins identified by Scaffold. Peptide Identifications were accepted if they could be established at greater than 91% probability to achieve an FDR less than 1.0% by the Scaffold Local FDR algorithm. Protein identifications were accepted if they could be established at greater than 52.0% probability to achieve an FDR less than 5.0% and contained at least 2 identified peptides.

^f^ Sequence coverage: percentage of amino acids of matched peptides in relation to the full sequence for each identified proteins.
