# Supplementary material for: Lys48 ubiquitination during the intraerythrocytic cycle of the rodent malaria parasite, Plasmodium chabaudi
Source: PLoS One. 2017 Jun 12;12(6):e0176533. doi: 10.1371/journal.pone.0176533 (PMC5467854; doi:10.1371/journal.pone.0176533)
Supplement: S2 Table — (DOCX) [file pone.0176533.s002.docx]

**Supplemental file 8. Ubiquitinated proteins found in other studies performed in *P. falciparum***

| Side code | Protein description | Found Ubiquitinated in other studies with  *P. falciparum* |
| --- | --- | --- |
| R1/S2 | Actin I | Wang L et al., 2015^1^  Ponts N et al., 2011^4^ |
| R2/T4/T5/S18 | Elongation Factor 1 alpha | Wang L et al., 2015^1^  Ponts N et al., 2011^4^ |
| T2/T3 | Glyceraldehyde-3-phosphate-dehydrogenase | Wang L et al., 2015^2^  Ponts N et al., 2011^4^ |
| S1 | Heat Shock Protein 70, putative | Wang L et al., 2015^1,2^  Ponts N et al., 2011^3^ |
| S3 | T-Complex protein 1 epsilon subunit | Wang L et al., 2015^1^  Ponts N et al., 2011^4^ |
| S4 | T-Complex protein subunit beta | Wang L et al., 2015^1^ |
| S8, S9, S10 | Purine nucleoside phosphorylase, putative (PNP) | Wang L et al., 2015^1^  Ponts N et al., 2011^4^ |
| S11 | Hypoxanthine-guanine-xanthine- phosphoribosyl transferase | Wang L et al., 2015^1^  Ponts N et al., 2011^4^ |
| S12 | Receptor for activated c kinase (RACK) | Wang L et al., 2015^1^  Ponts N et al., 2011^4^ |
| S13 | Phosphoglycerate kinase OS | Wang L et al., 2015^1^  Ponts N et al., 2011^4^ |
| S14 | Hexokinase OS | Wang L et al., 2015^1^  Ponts N et al., 2011^4^ |
| S15 | Pyruvate kinase | Wang L et al., 2015^1^  Ponts N et al., 2011^4^ |
| S16 | Inosine-5'-monophosphate dehydrogenase | Wang L et al., 2015^1^  Ponts N et al., 2011^4^ |
| S17 | Hsp70/Hsp90 organizing protein, putative (HOP) | Wang L et al., 2015^1^ |
| S19 | Proliferation associated protein 2g4, putative | Wang L et al., 2015^1^  Ponts N et al., 2011^4^ |

^1^Co-identified proteins in the affinity purification of *P. falciparum* 26S proteasomes

^2^Identified proteins in GST-based mock purifications of the 26S proteasome of *P. falciparum*

^3^ Putative Plasmodium ubiquitin conjugates (significantly enriched compared to negative controls, i.e. with p-values <0.05 in at least one of the 3 blood stages examined)

^4^ Plasmodium proteins detected in both specific IPs and negative controls (not specifically enriched in Ub IPs)
