# Supplementary material for: Lys48 ubiquitination during the intraerythrocytic cycle of the rodent malaria parasite, Plasmodium chabaudi
Source: PLoS One. 2017 Jun 12;12(6):e0176533. doi: 10.1371/journal.pone.0176533 (PMC5467854; doi:10.1371/journal.pone.0176533)
Supplement: S2 Fig — (A) Sequence of the ORF of the polyubiquitin gene (B) Alignment of the DNA sequence of the four tandem repeats of the polyubiquitin gene using Clustal Omega (C) Alignment of the aminoacids sequence of the four tandem repeats of the Ubiquitin polypeptides using Clustal Omega. (DOCX) [file pone.0176533.s004.docx]

**A)**

>Forward

ATGCAAATCTTTGTGAAAACATTAACAGGAAAAACTATAACCCTTGATGTTGAGCCATCCGATACCATTGAAAATGTTAAAGCTAAAATTCAAGATAAAGAAGGTATCCCACCTGATCAACAAAGATTAATTTTTGCTGGAAAGCAATTAGAAGATGGAAGAACATTATCTGACTATAACATTCAAAAAGAATCTACCTTACACTTAGTTTTAAGATTAAGAGGAGGTATGCAGATTTTCGTAAAAACTTTAACCGGGAAAACAATTACTCTTGATGTTGAACCATCTGATACTATTGAAAATGTTAAAGCTAAAATTCAAGATAAAGAAGGTATTCCACCTGATCAACAAAGATTAATTTTCGCTGGAAAACAATTAGAGGATGGAAGAACACTATCTGATTACAACATTCAAAAAGAATCTACCTTACACTTAGTTTTAAGATTAAGAGGAGGTATGCAGATTTTCGTAAAAACTTTAACCGGGAAAACAATCACTCTTGATGTTGAGCCATCTGATACCATTGAAAATGTTAAAGCTAAAATTCAAGATAAAGAAGGTATTCCTCCTGATCAACAAAGATTAATTTTTGCCGGAAAACAATTAGAAGATGGTAGAACACTATCTGACTACAACATTCAAAAAGAATCGACTTTACACTTAGTTTTAAGATTAAGAGGAGGTATGCAAATTTTCGTAAAAACATTAACCGGAAAAACAATTACTCTTGATGTTGAACCATCAGATACTATTGAAAACGTTAAAGCCAAAATTCAAGATAAAGAAGGTATCCCACCTGATCAACAAAGATTAATTTTCGCTGGAAAACAATTAGAAGACGGTAGAACATTATCTGACTATAACATTCAAAAAGAATCTACCTTACATTTAGTTTTAAGATTAAGAGGAGGTTGCTAA

>Reverse

TTAGCAACCTCCTCTTAATCTTAAAACTAAATGTAAGGTAGATTCTTTTTGAATGTTATAGTCAGATAATGTTCTACCGTCTTCTAATTGTTTTCCAGCGAAAATTAATCTTTGTTGATCAGGTGGGATACCTTCTTTATCTTGAATTTTGGCTTTAACGTTTTCAATAGTATCTGATGGTTCAACATCAAGAGTAATTGTTTTTCCGGTTAATGTTTTTACGAAAATTTGCATACCTCCTCTTAATCTTAAAACTAAGTGTAAAGTCGATTCTTTTTGAATGTTGTAGTCAGATAGTGTTCTACCATCTTCTAATTGTTTTCCGGCAAAAATTAATCTTTGTTGATCAGGAGGAATACCTTCTTTATCTTGAATTTTAGCTTTAACATTTTCAATGGTATCAGATGGCTCAACATCAAGAGTGATTGTTTTCCCGGTTAAAGTTTTTACGAAAATCTGCATACCTCCTCTTAATCTTAAAACTAAGTGTAAGGTAGATTCTTTTTGAATGTTGTAATCAGATAGTGTTCTTCCATCCTCTAATTGTTTTCCAGCGAAAATTAATCTTTGTTGATCAGGTGGAATACCTTCTTTATCTTGAATTTTAGCTTTAACATTTTCAATAGTATCAGATGGTTCAACATCAAGAGTAATTGTTTTCCCGGTTAAAGTTTTTACGAAAATCTGCATACCTCCTCTTAATCTTAAAACTAAGTGTAAGGTAGATTCTTTTTGAATGTTATAGTCAGATAATGTTCTTCCATCTTCTAATTGCTTTCCAGCAAAAATTAATCTTTGTTGATCAGGTGGGATACCTTCTTTATCTTGAATTTTAGCTTTAACATTTTCAATGGTATCGGATGGCTCAACATCAAGGGTTATAGTTTTTCCTGTTAATGTTTTCACAAAGATTTGCAT

**B)**

Ub_Repeat_1 ATGCAAATCTTTGTGAAAACATTAACAGGAAAAACTATAACCCTTGATGTTGAGCCATCC

Ub_Repeat_2 ATGCAGATTTTCGTAAAAACTTTAACCGGGAAAACAATTACTCTTGATGTTGAACCATCT

Ub_Repeat_3 ATGCAGATTTTCGTAAAAACTTTAACCGGGAAAACAATCACTCTTGATGTTGAGCCATCT

Ub_Repeat_4 ATGCAAATTTTCGTAAAAACATTAACCGGAAAAACAATTACTCTTGATGTTGAACCATCA

***** ** ** ** ***** ***** ** ***** ** ** *********** *****

Ub_Repeat_1 GATACCATTGAAAATGTTAAAGCTAAAATTCAAGATAAAGAAGGTATCCCACCTGATCAA

Ub_Repeat_2 GATACTATTGAAAATGTTAAAGCTAAAATTCAAGATAAAGAAGGTATTCCACCTGATCAA

Ub_Repeat_3 GATACCATTGAAAATGTTAAAGCTAAAATTCAAGATAAAGAAGGTATTCCTCCTGATCAA

Ub_Repeat_4 GATACTATTGAAAACGTTAAAGCCAAAATTCAAGATAAAGAAGGTATCCCACCTGATCAA

***** ******** ******** *********************** ** *********

Ub_Repeat_1 CAAAGATTAATTTTTGCTGGAAAGCAATTAGAAGATGGAAGAACATTATCTGACTATAAC

Ub_Repeat_2 CAAAGATTAATTTTCGCTGGAAAACAATTAGAGGATGGAAGAACACTATCTGATTACAAC

Ub_Repeat_3 CAAAGATTAATTTTTGCCGGAAAACAATTAGAAGATGGTAGAACACTATCTGACTACAAC

Ub_Repeat_4 CAAAGATTAATTTTCGCTGGAAAACAATTAGAAGACGGTAGAACATTATCTGACTATAAC

************** ** ***** ******** ** ** ****** ******* ** ***

Ub_Repeat_1 ATTCAAAAAGAATCTACCTTACACTTAGTTTTAAGATTAAGAGGAGGT------

Ub_Repeat_2 ATTCAAAAAGAATCTACCTTACACTTAGTTTTAAGATTAAGAGGAGGT------

Ub_Repeat_3 ATTCAAAAAGAATCGACTTTACACTTAGTTTTAAGATTAAGAGGAGGT------

Ub_Repeat_4 ATTCAAAAAGAATCTACCTTACATTTAGTTTTAAGATTAAGAGGAGGTTGCTAA

************** ** ***** ************************

**C)**

Ub_Repeat_1 MQIFVKTLTGKTITLDVEPSDTIENVKAKIQDKEGIPPDQQRLIFAGKQLEDGRTLSDYNIQKESTLHLVLRLRGG-

Ub_Repeat_2 MQIFVKTLTGKTITLDVEPSDTIENVKAKIQDKEGIPPDQQRLIFAGKQLEDGRTLSDYNIQKESTLHLVLRLRGG-

Ub_Repeat_3 MQIFVKTLTGKTITLDVEPSDTIENVKAKIQDKEGIPPDQQRLIFAGKQLEDGRTLSDYNIQKESTLHLVLRLRGG-

Ub_Repeat_4 MQIFVKTLTGKTITLDVEPSDTIENVKAKIQDKEGIPPDQQRLIFAGKQLEDGRTLSDYNIQKESTLHLVLRLRGGC

****************************************************************************
