# Supplementary figures and images for: Lys48 ubiquitination during the intraerythrocytic cycle of the rodent malaria parasite, Plasmodium chabaudi
Source: PLoS One. 2017 Jun 12;12(6):e0176533. doi: 10.1371/journal.pone.0176533 (PMC5467854; doi:10.1371/journal.pone.0176533)

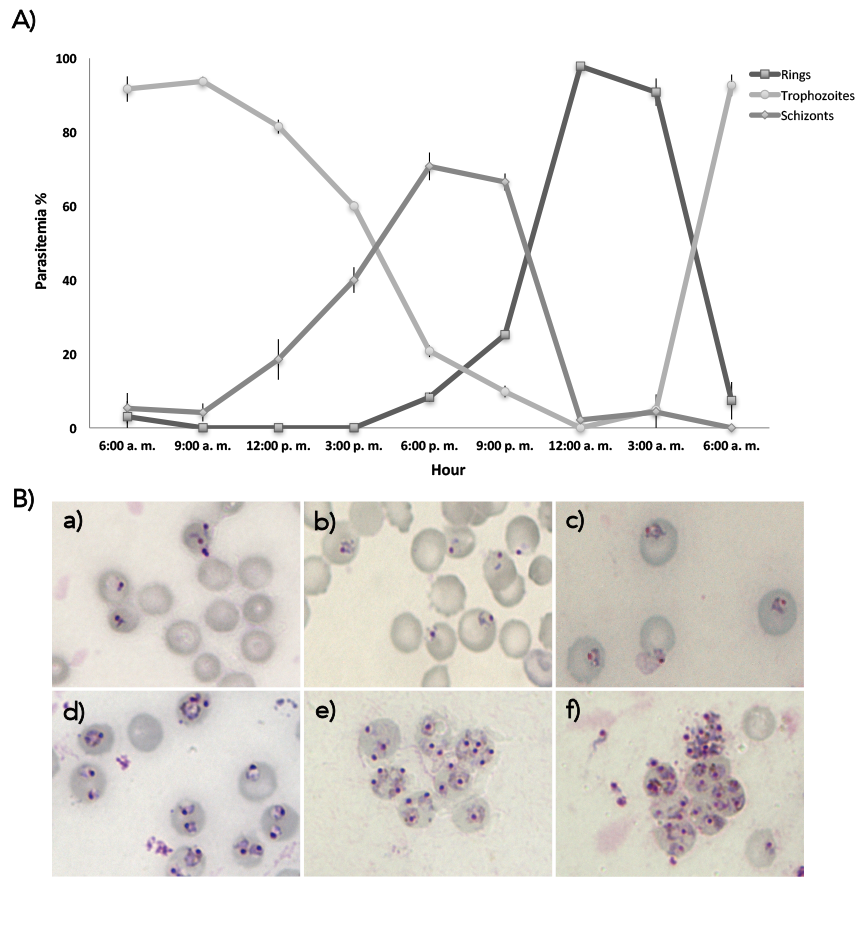

Supplement: S3 Fig — A) Male BALB/c mice of 6–8 weeks were infected with 1 x 10^6^ parasites. Every 3 hours the parasite stages were counted. Counts were performed by triplicates, and are expressed as percentage of infected erythrocytes. B) Different intraerythrocytic stages of P. chabaudi obtained after Percoll purification. Smears of the parasites purified with Percoll-Sucrose gradients were stained with 20% Giemsa. a) Ring stage (3 am); b-d Trophozoite stage (obtained at 9 am, 3 pm, 7 pm, respectively); e-f) Schizont stage (obtained at 6 pm and 9 pm, respectively). (TIF) [file pone.0176533.s005.tif]

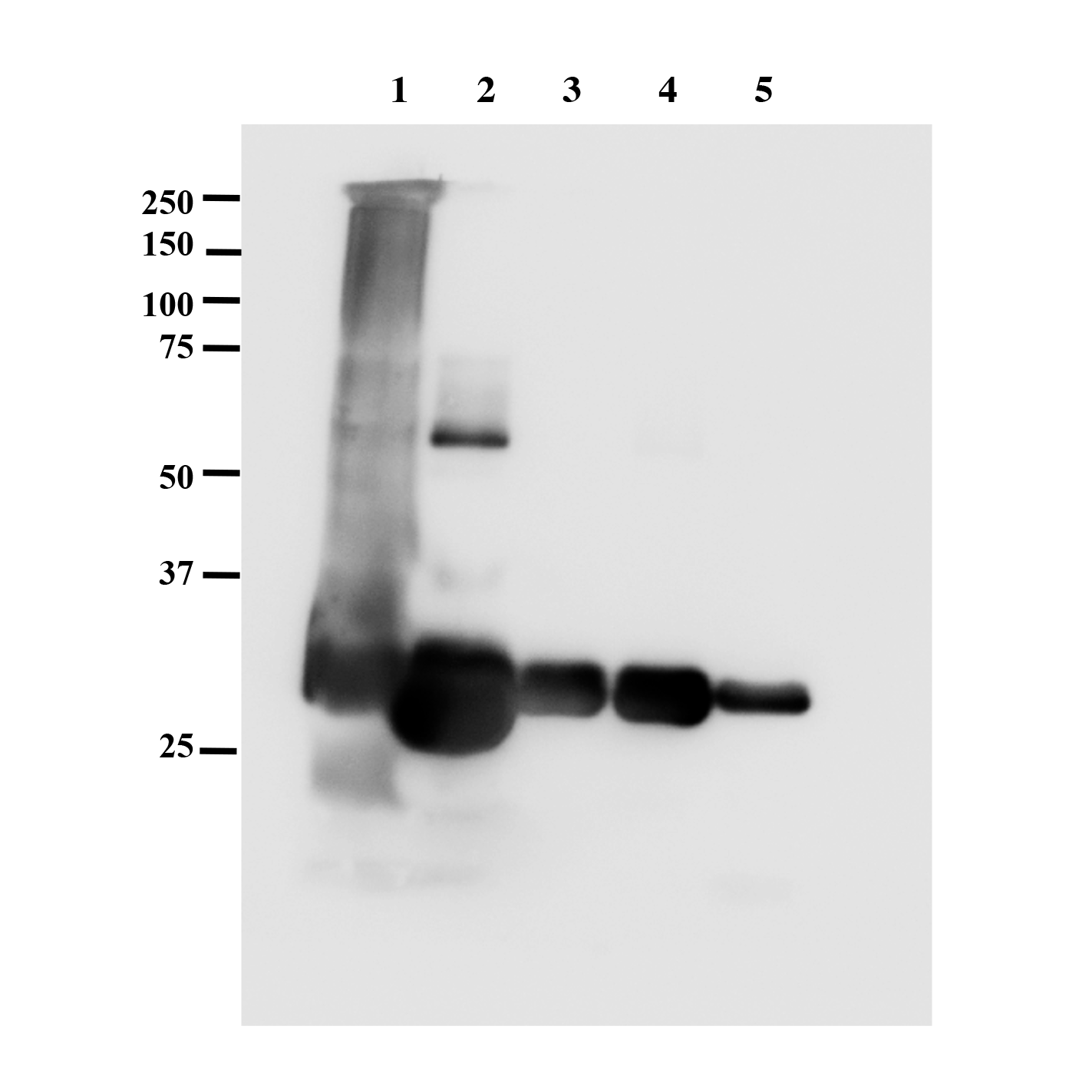

Supplement: S4 Fig — Specificity of the anti-Ubiquitin monoclonal antibody was tested with the Ubiquitin monomer recombinant protein of P. chabaudi. Lane 1–5. Successive fractions after chromatographic elution of GST-PcUb. (TIF) [file pone.0176533.s006.tif]

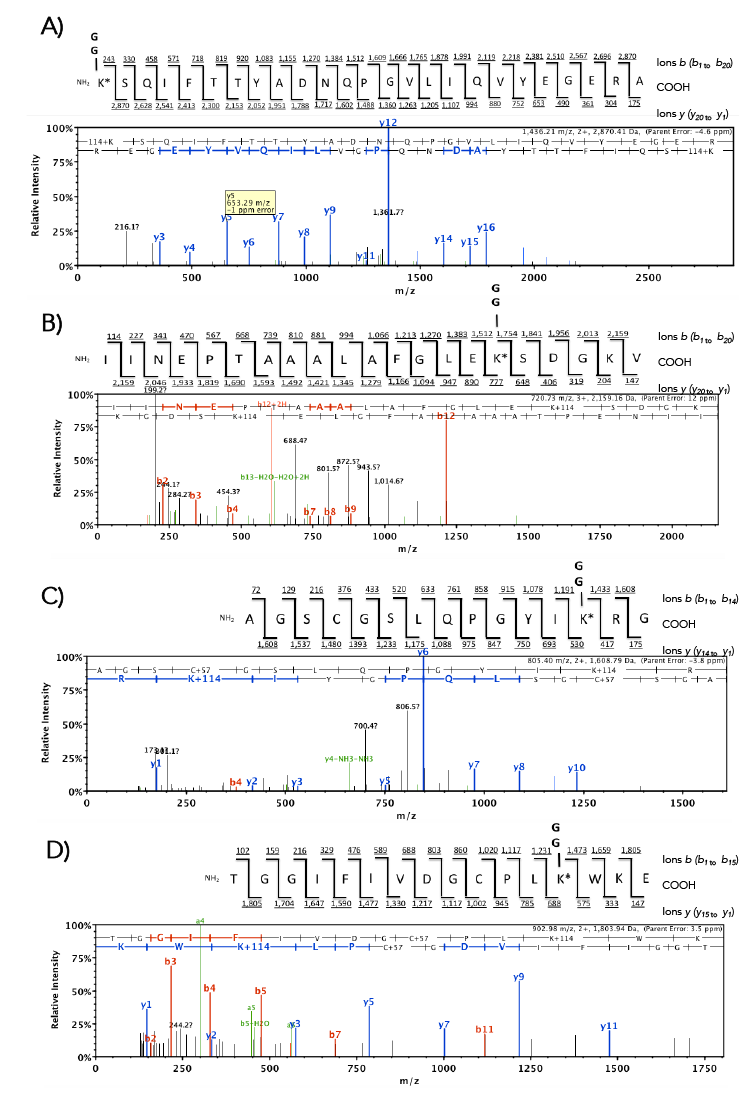

Supplement: S6 Fig — After trypsin digestion, the peptide that contains the post-translational modification has a diglycline remnant covalently attached to a lysine residue that is resistant to the trypsin proteolysis (36). On each figure (A-D) is shown the peptide sequence produced by the trypsin proteolysis with the ion mass (type b and y ions) and with the diglycine modification (top figure) and the fragmentation pattern (MS/MS spectra) acquired for the peptide sequence construction. (A) Identification of the lysine modification for a heat-shock protein, putative; (B) Identification of the lysine modification for a heat-shock protein 70; (C-D) Identification of the lysines modifications for the uridine phosphorylase/purine nucleoside phosphorylase. (TIF) [file pone.0176533.s008.tif]
